# Correction: Segmentation of HE-stained meningioma pathological images based on pseudo-labels

**DOI:** 10.1371/journal.pone.0314703

**Published:** 2024-11-26

**Authors:** Chongshu Wu, Jing Zhong, Lin Lin, Yanping Chen, Yunjing Xue, Peng Shi

There are errors in the author affiliations. The correct affiliations are as follows:

Chongshu Wu^1,5^, Jing Zhong^2^, Lin Lin^3^, Yanping Chen^4^, Yunjing Xue^3^, Peng Shi^1,5^

**1** College of Computer and Cyber Security, Fujian Normal University, Fuzhou, Fujian, China, **2** Department of Radiology, Fujian Medical University Cancer Hospital, Fujian Cancer Hospital, Fuzhou, Fujian, China, **3** Radiology Department, Fujian Medical University Union Hospital, Fuzhou, Fujian, China, **4** Department of Pathology, Fujian Medical University Cancer Hospital, Fujian Cancer Hospital, Fuzhou, Fujian, China, **5** Digit Fujian Internet-of-Things Laboratory of Environmental Monitoring, Fuzhou, Fujian, China.
