# Supplementary figures and images for: Segmentation of HE-stained meningioma pathological images based on pseudo-labels
Source: PLoS One. 2022 Feb 4;17(2):e0263006. doi: 10.1371/journal.pone.0263006 (PMC8815980; doi:10.1371/journal.pone.0263006)

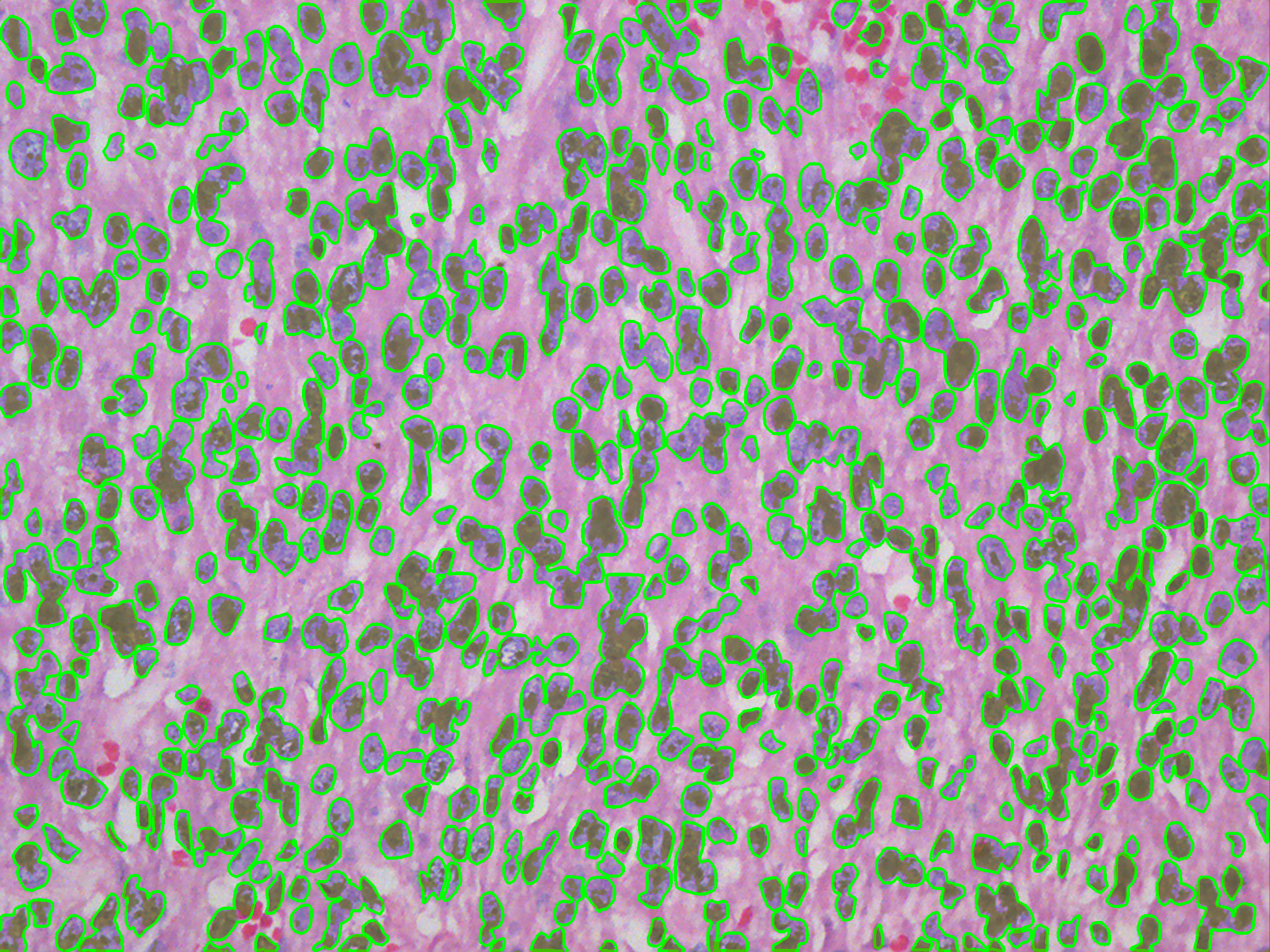

Supplement: S1 Fig — (ZIP) [file pone.0263006.s001.zip › Fig 2/Complete Image 1.jpg]

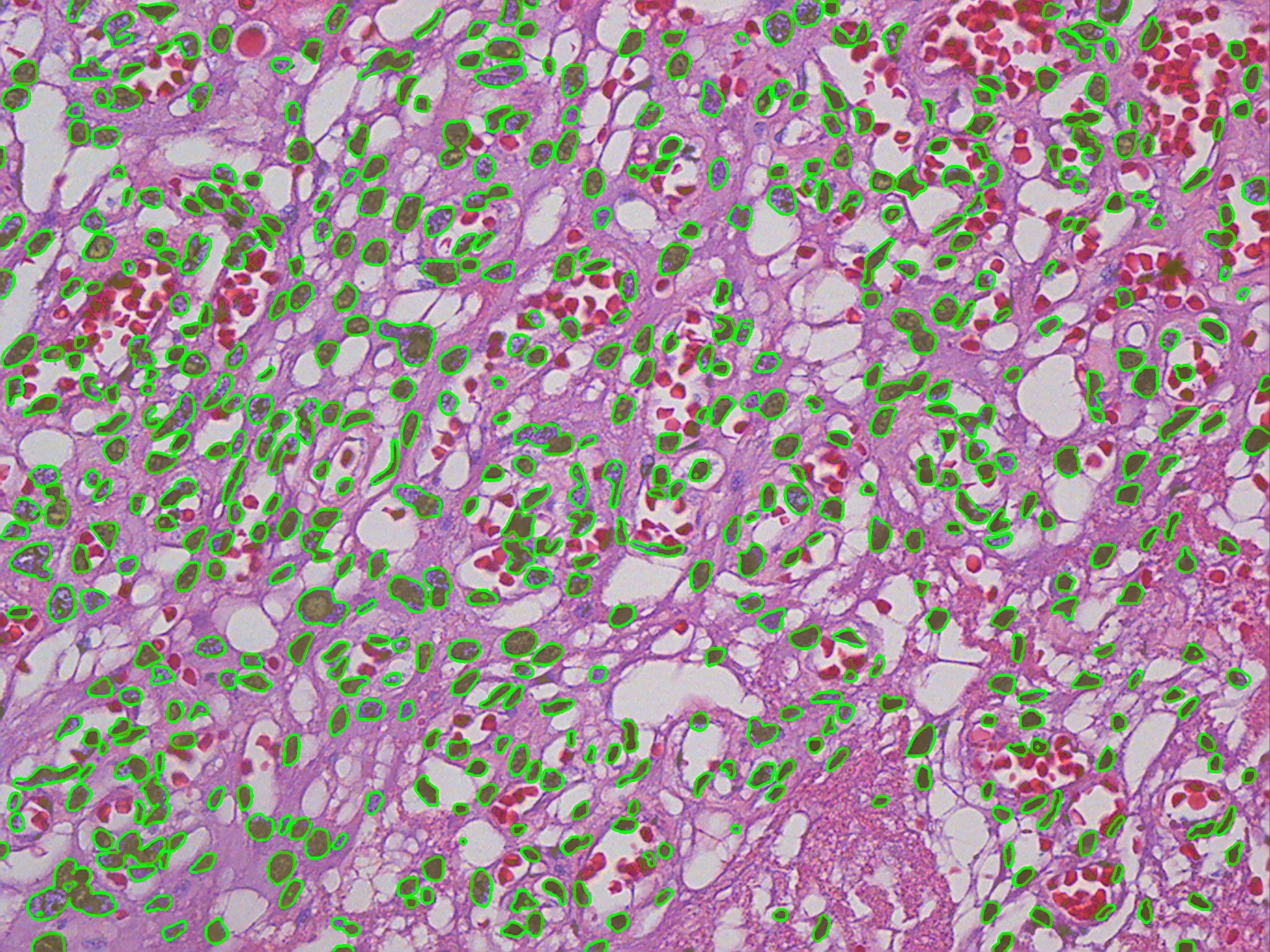

Supplement: S1 Fig — (ZIP) [file pone.0263006.s001.zip › Fig 2/Complete Image 2.jpg]

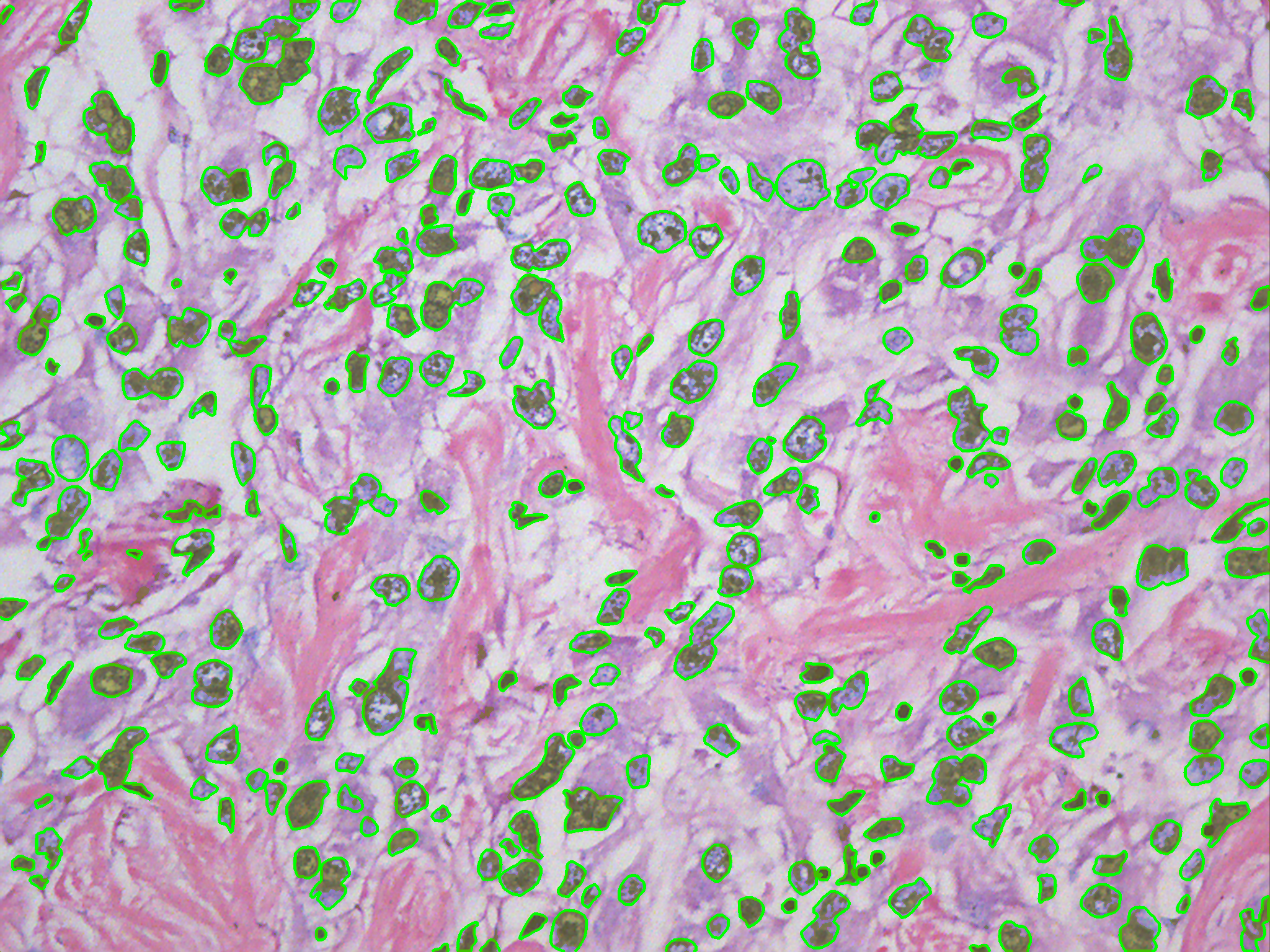

Supplement: S1 Fig — (ZIP) [file pone.0263006.s001.zip › Fig 2/Complete Image 3.jpg]

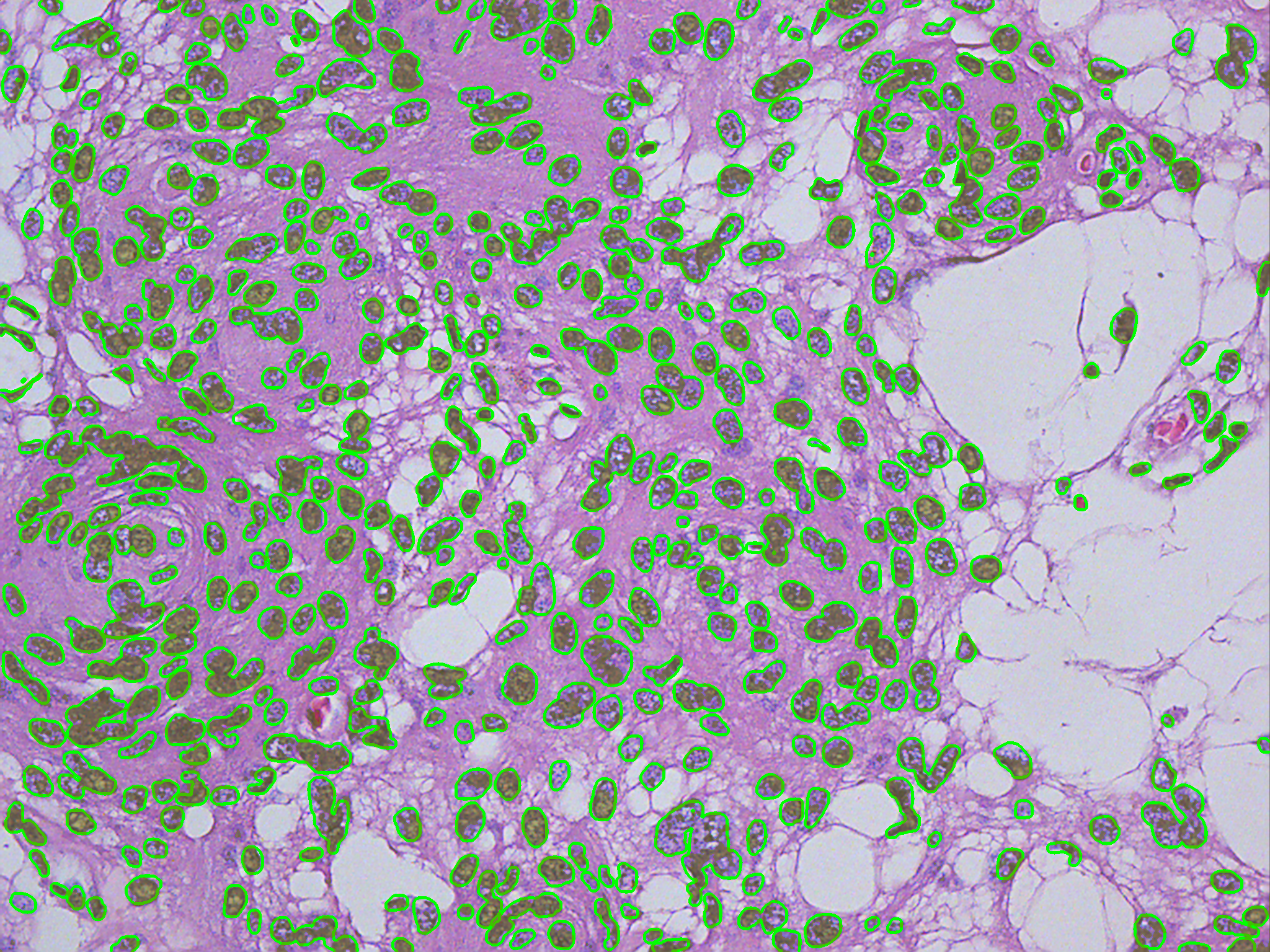

Supplement: S1 Fig — (ZIP) [file pone.0263006.s001.zip › Fig 2/Complete Image 4.jpg]

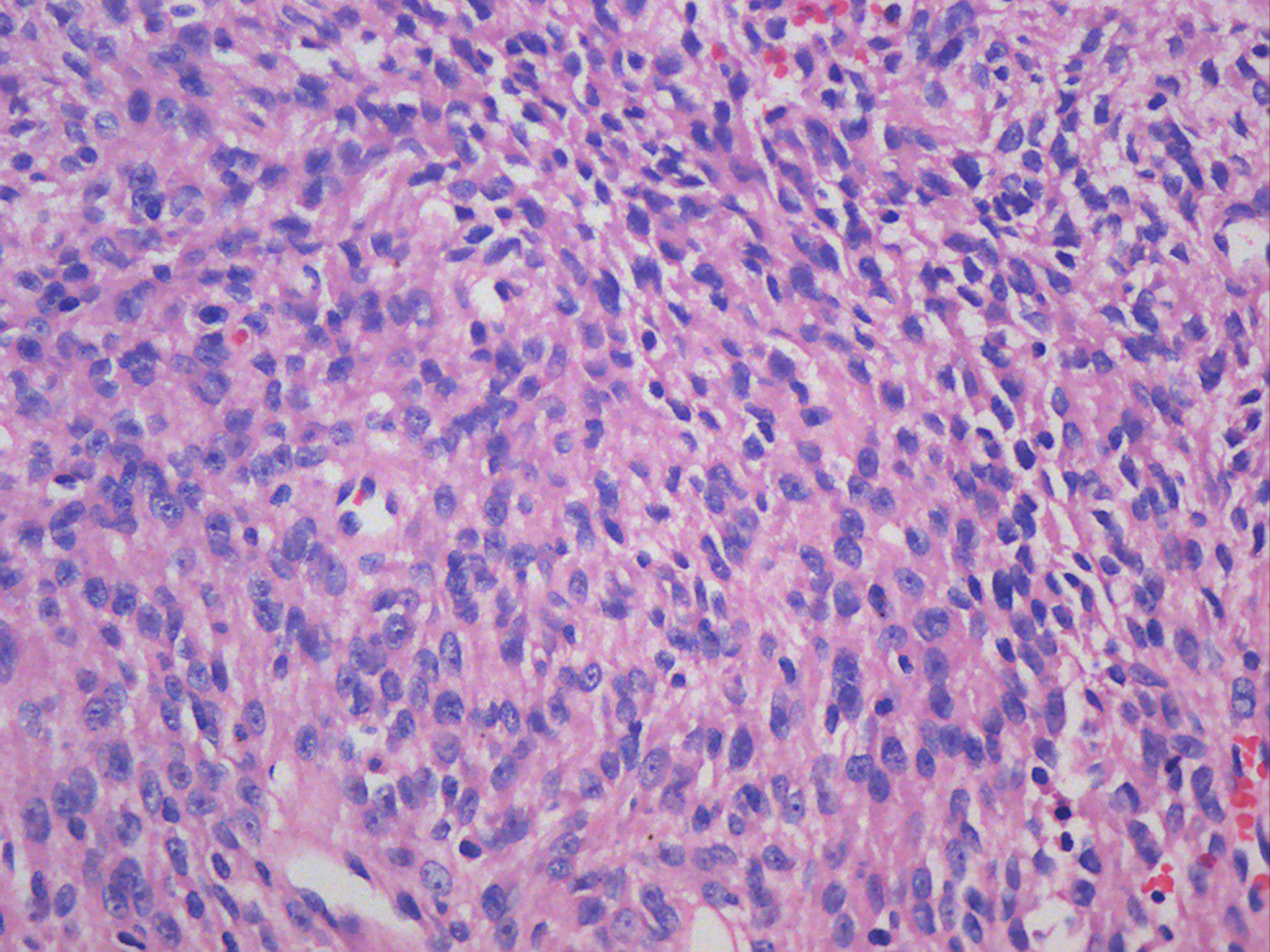

Supplement: S1 Fig — (ZIP) [file pone.0263006.s001.zip › Fig 2/Original HE Image 1.tif]

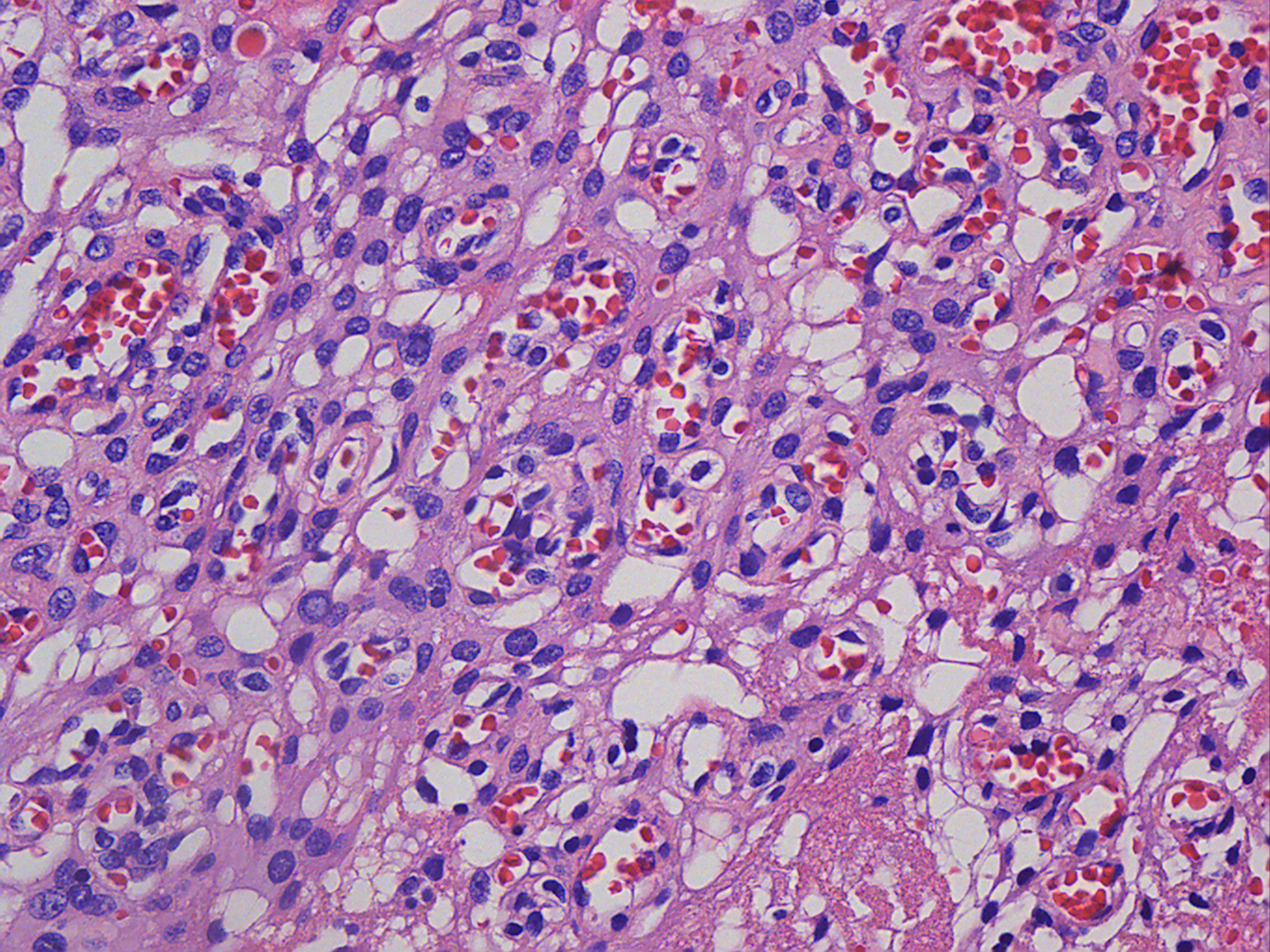

Supplement: S1 Fig — (ZIP) [file pone.0263006.s001.zip › Fig 2/Original HE Image 2.tif]

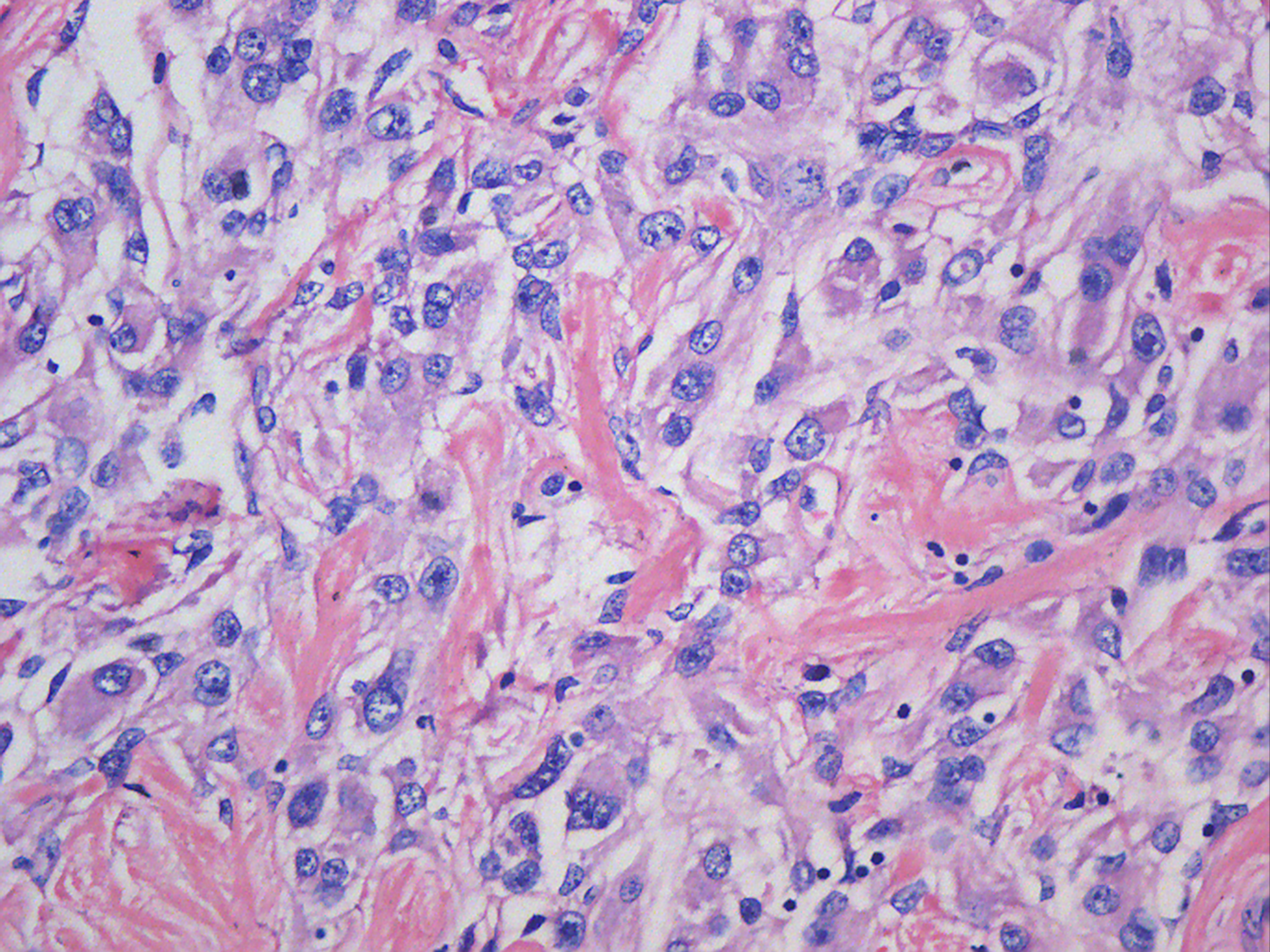

Supplement: S1 Fig — (ZIP) [file pone.0263006.s001.zip › Fig 2/Original HE Image 3.tif]

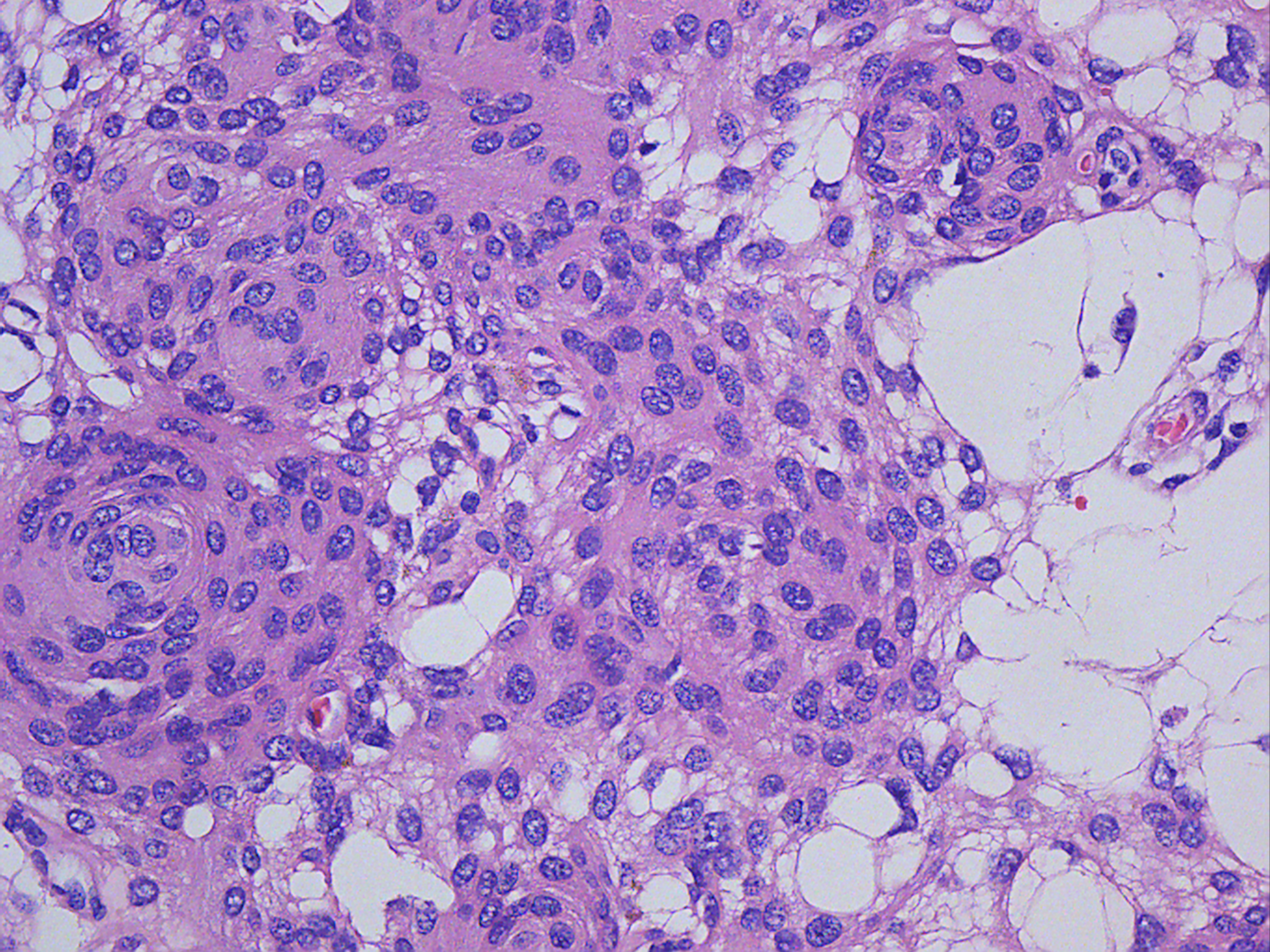

Supplement: S1 Fig — (ZIP) [file pone.0263006.s001.zip › Fig 2/Original HE Image 4.tif]

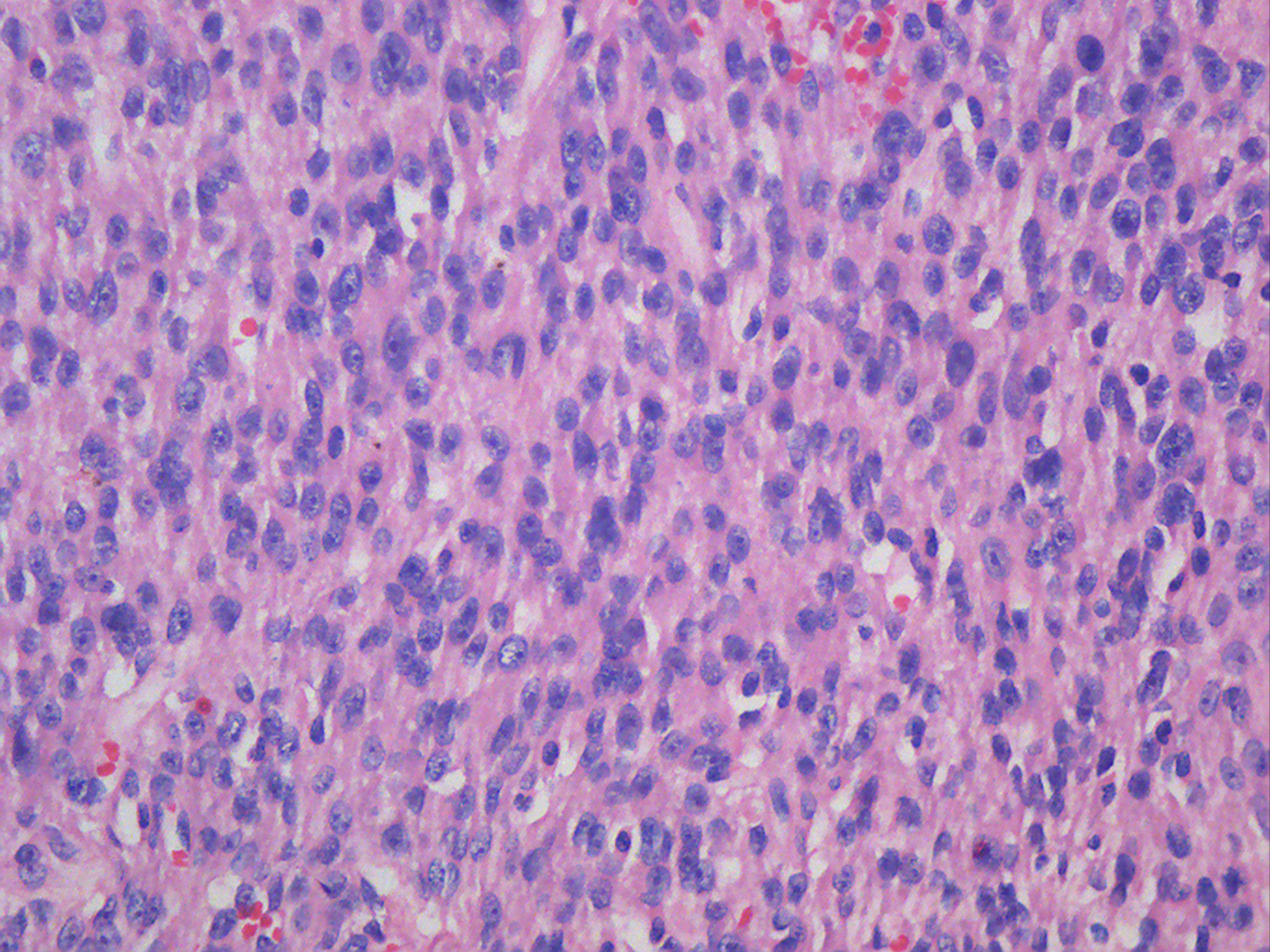

Supplement: S1 Fig — (ZIP) [file pone.0263006.s001.zip › Fig 4/Original Image 1.tif]

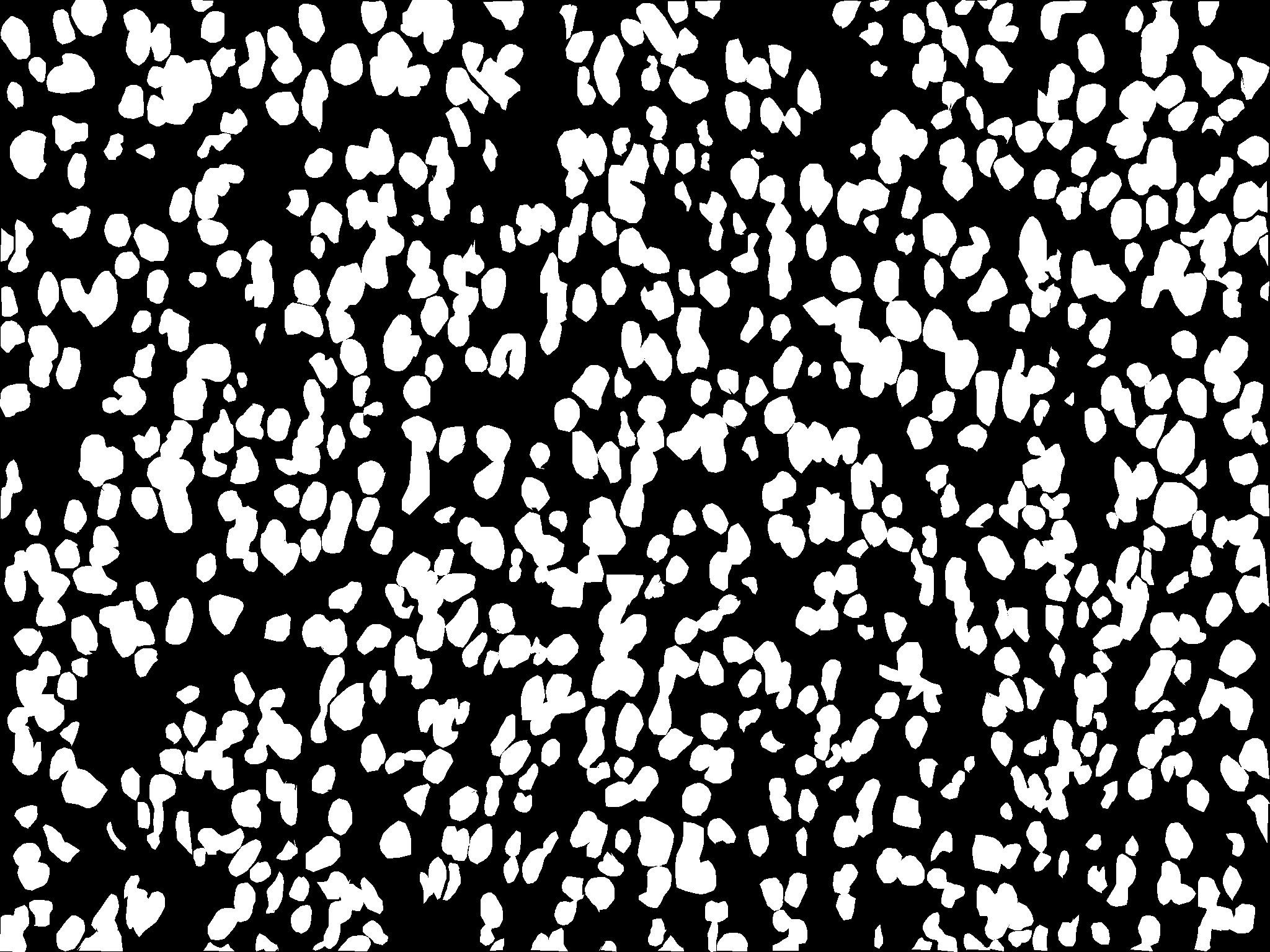

Supplement: S1 Fig — (ZIP) [file pone.0263006.s001.zip › Fig 4/Original Label 1.jpg]

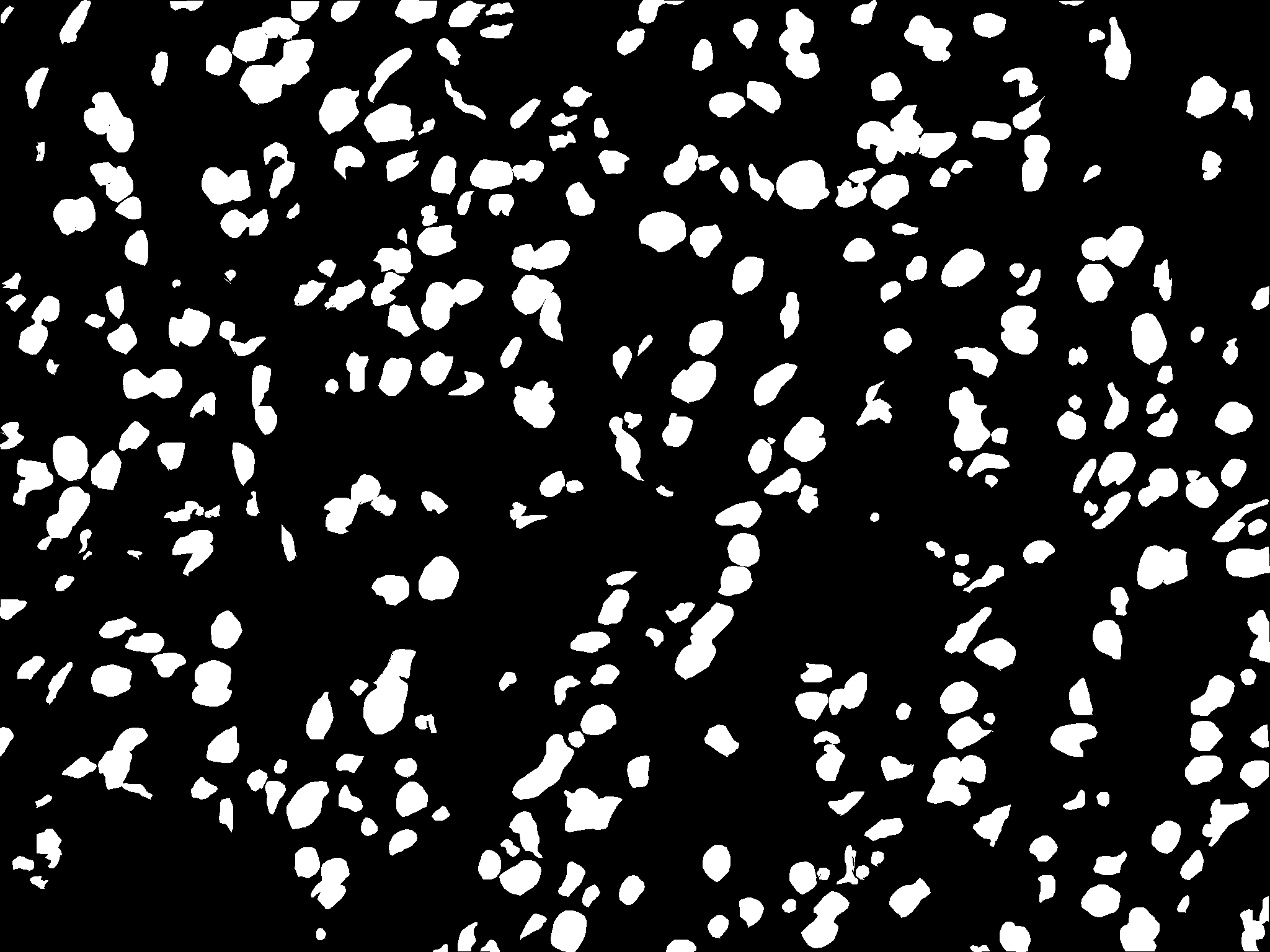

Supplement: S1 Fig — (ZIP) [file pone.0263006.s001.zip › Fig 4/Original Label 2.jpg]

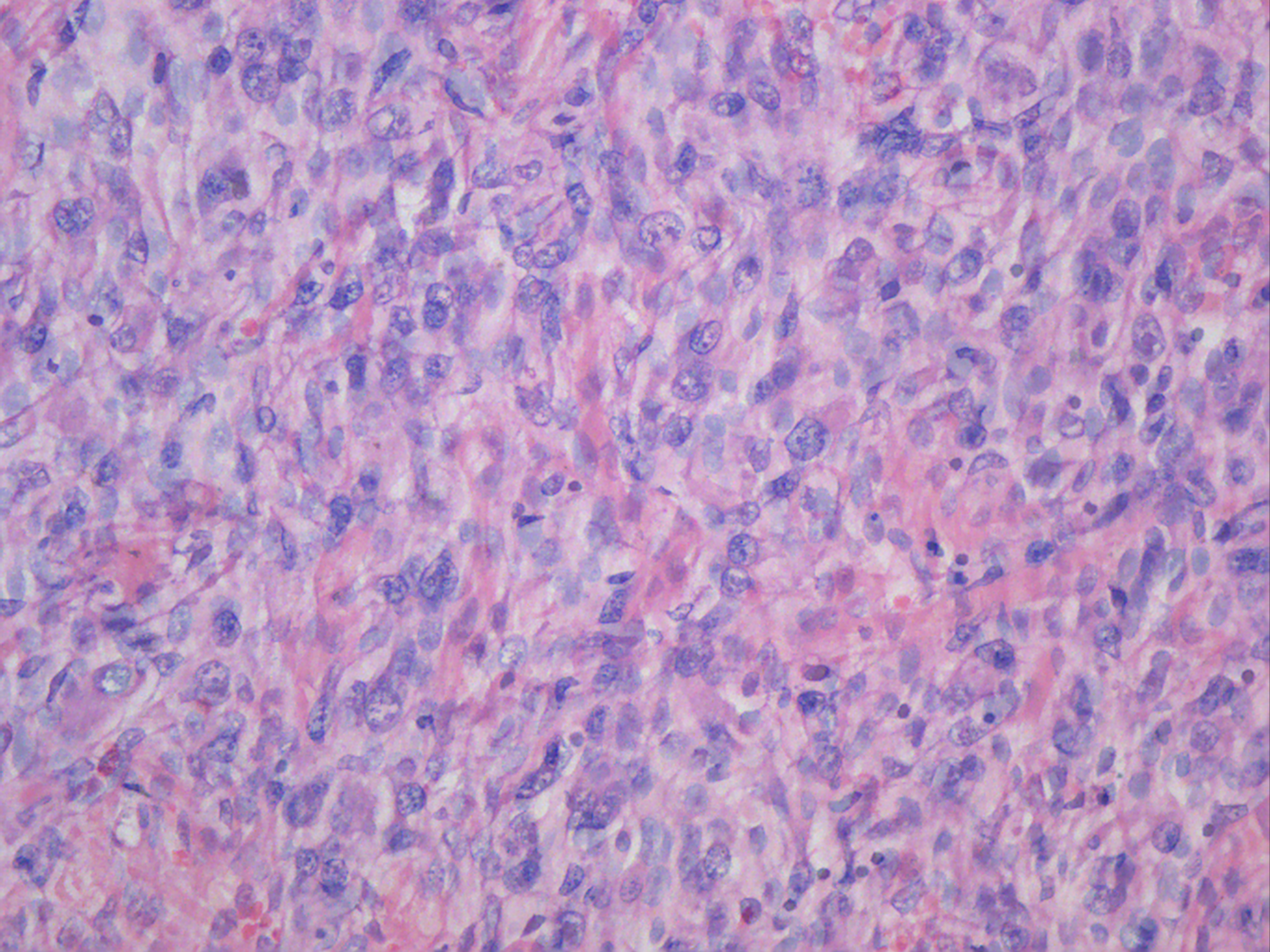

Supplement: S1 Fig — (ZIP) [file pone.0263006.s001.zip › Fig 4/Original Mixup Image.jpg]

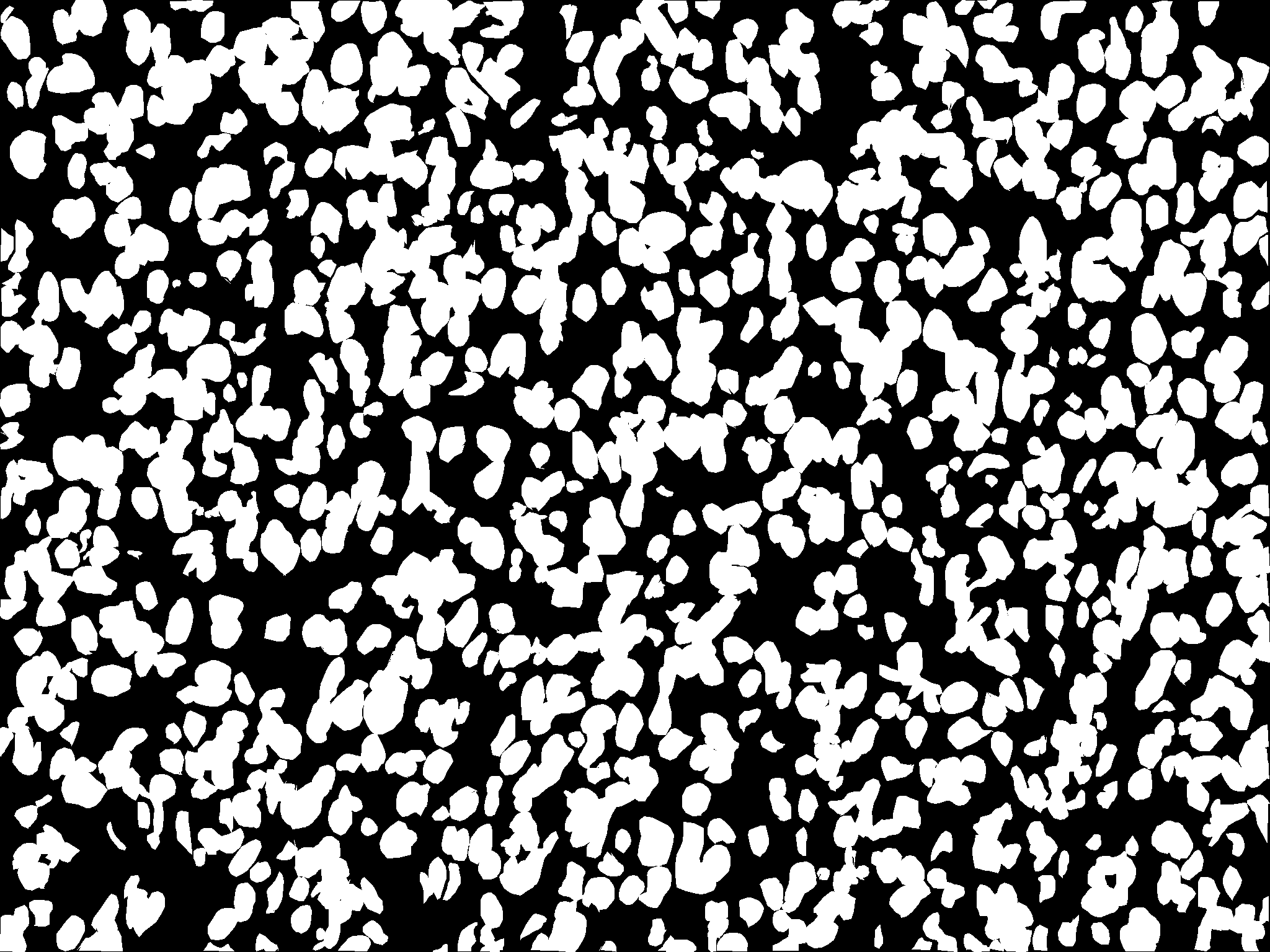

Supplement: S1 Fig — (ZIP) [file pone.0263006.s001.zip › Fig 4/Original Mixup Label.jpg]

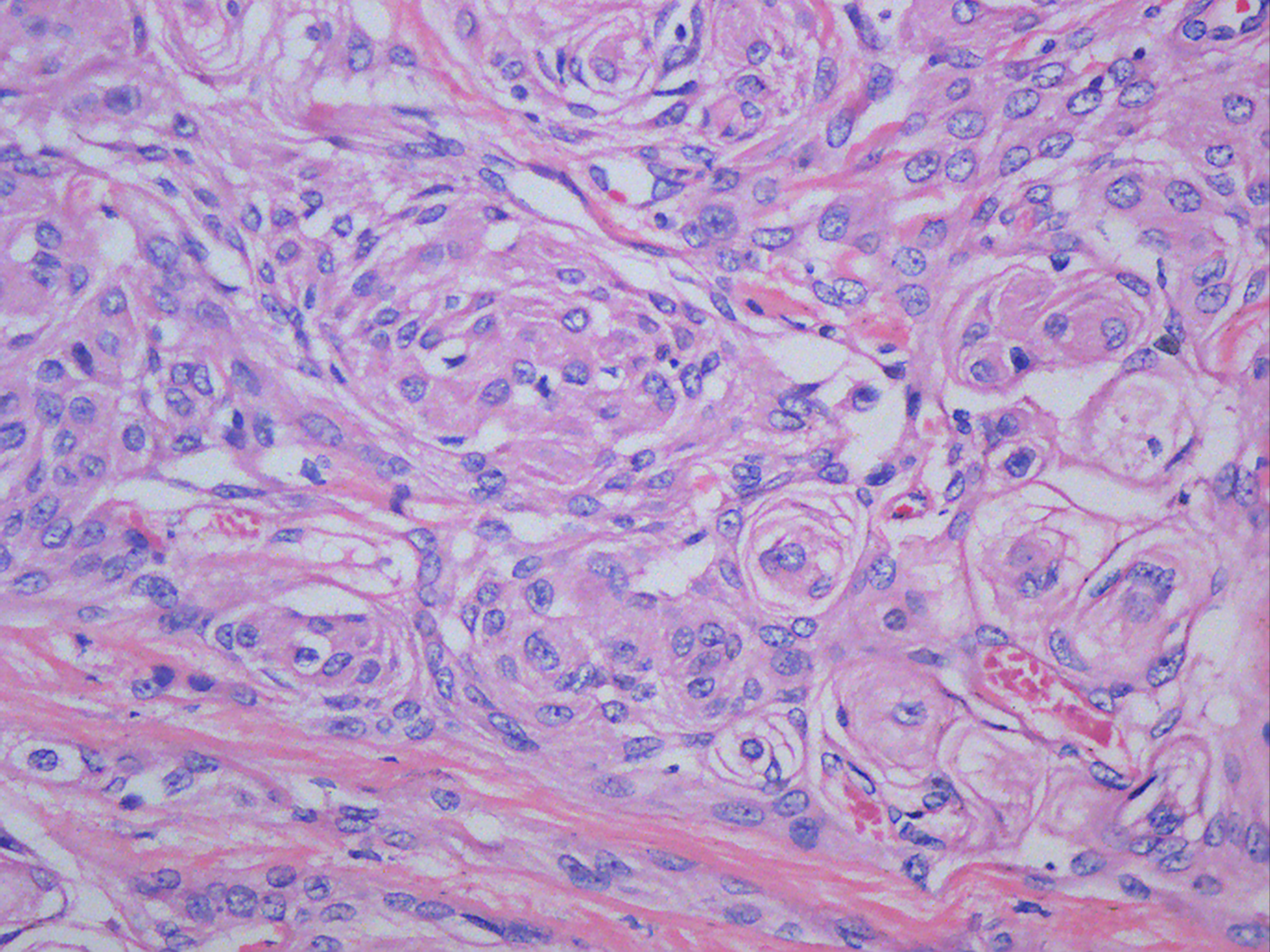

Supplement: S2 Fig — (ZIP) [file pone.0263006.s002.zip › Original High-grade Image 3.bmp]

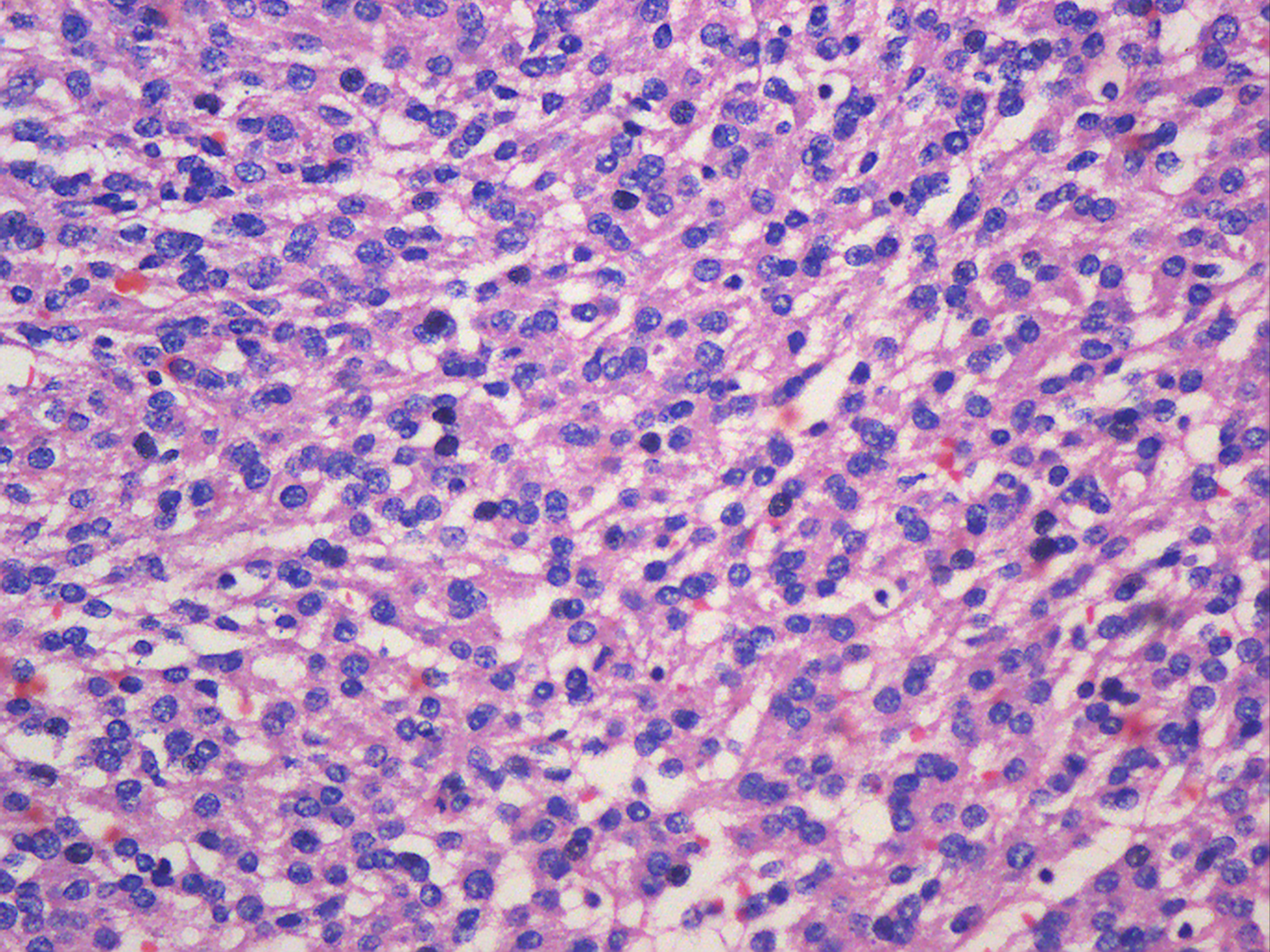

Supplement: S2 Fig — (ZIP) [file pone.0263006.s002.zip › Original High-grade Image 4.bmp]

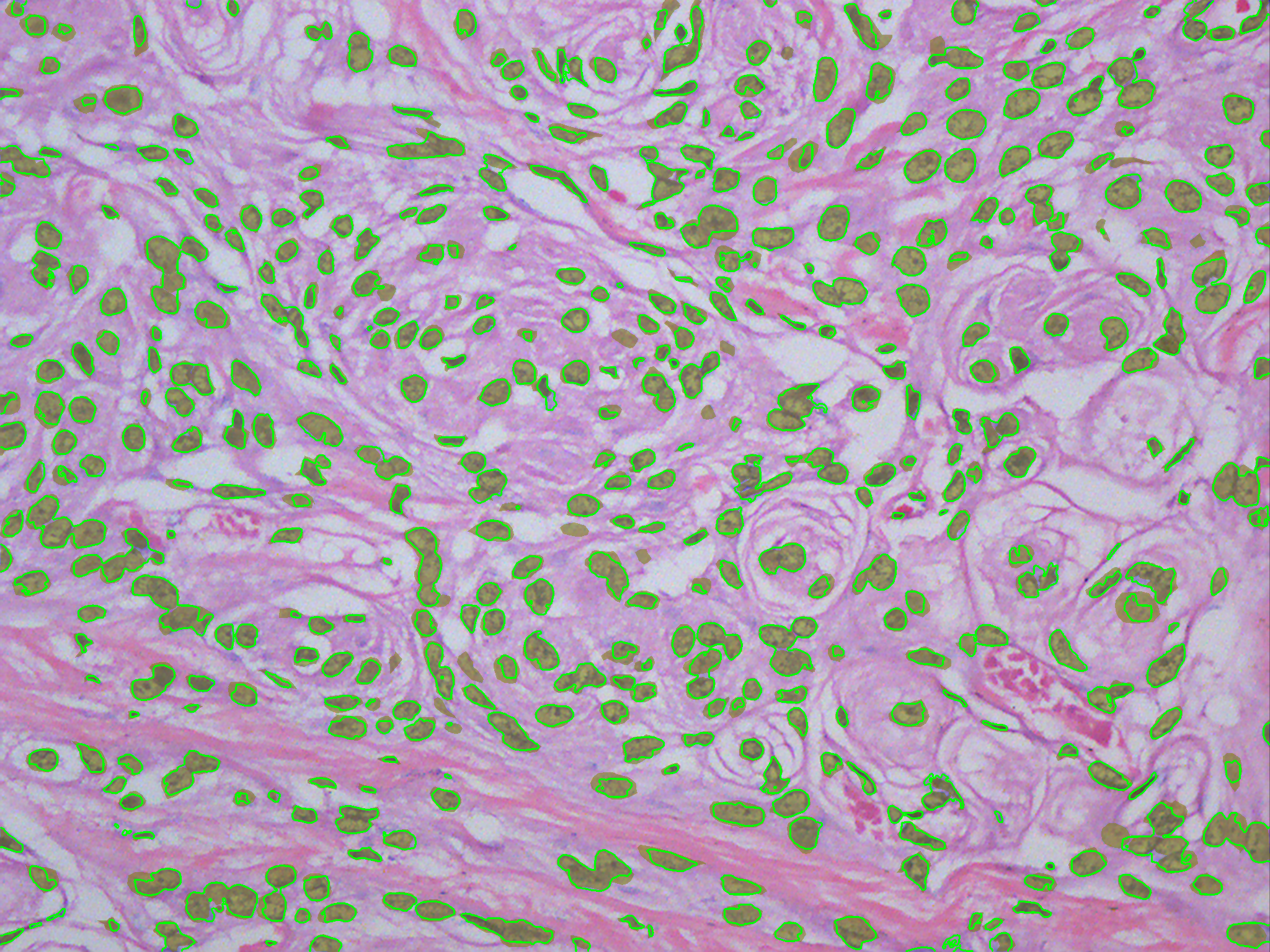

Supplement: S2 Fig — (ZIP) [file pone.0263006.s002.zip › Original High-grade Segmentation Result 3.jpg]

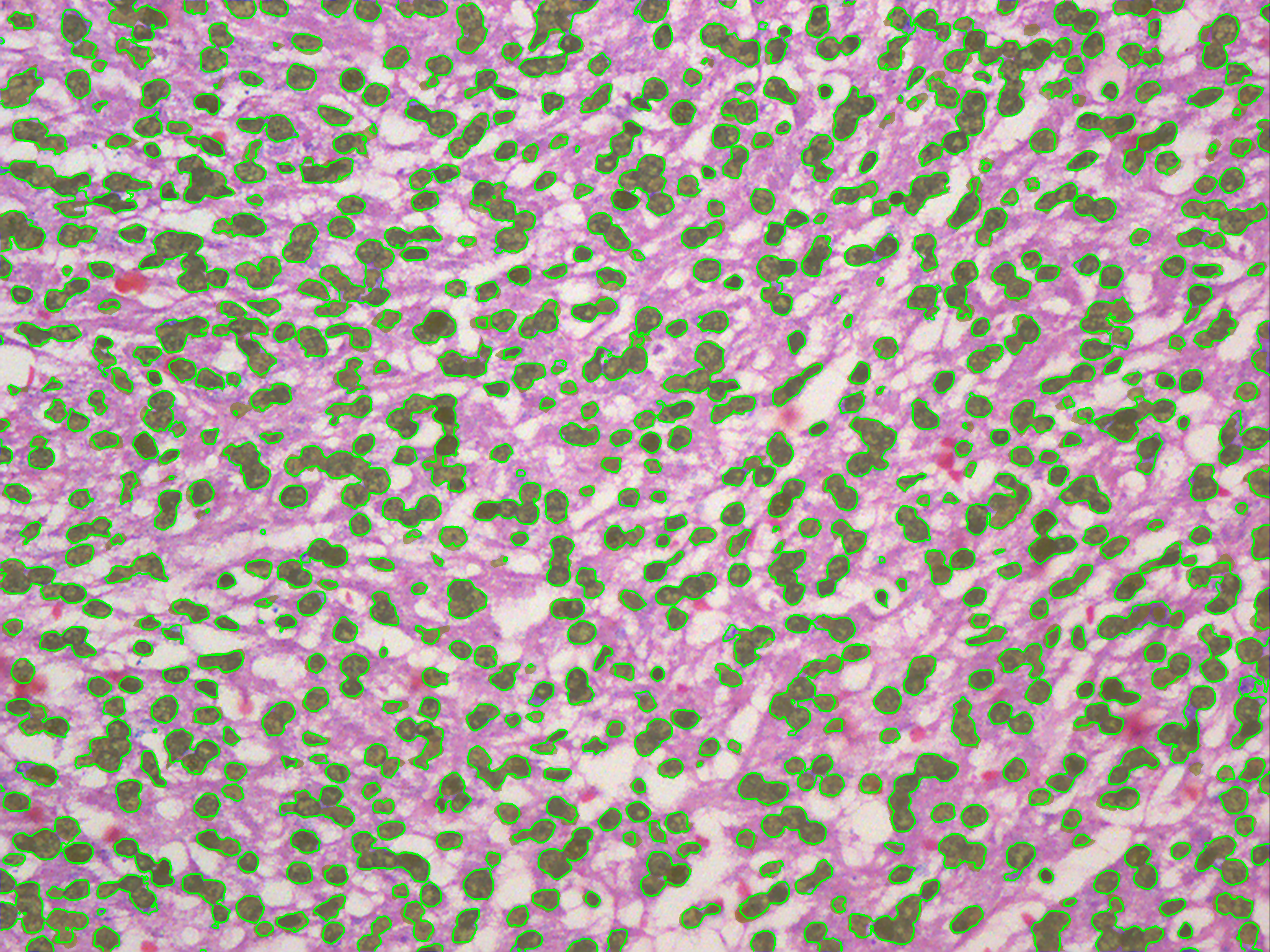

Supplement: S2 Fig — (ZIP) [file pone.0263006.s002.zip › Original High-grade Segmentation Result 4.jpg]

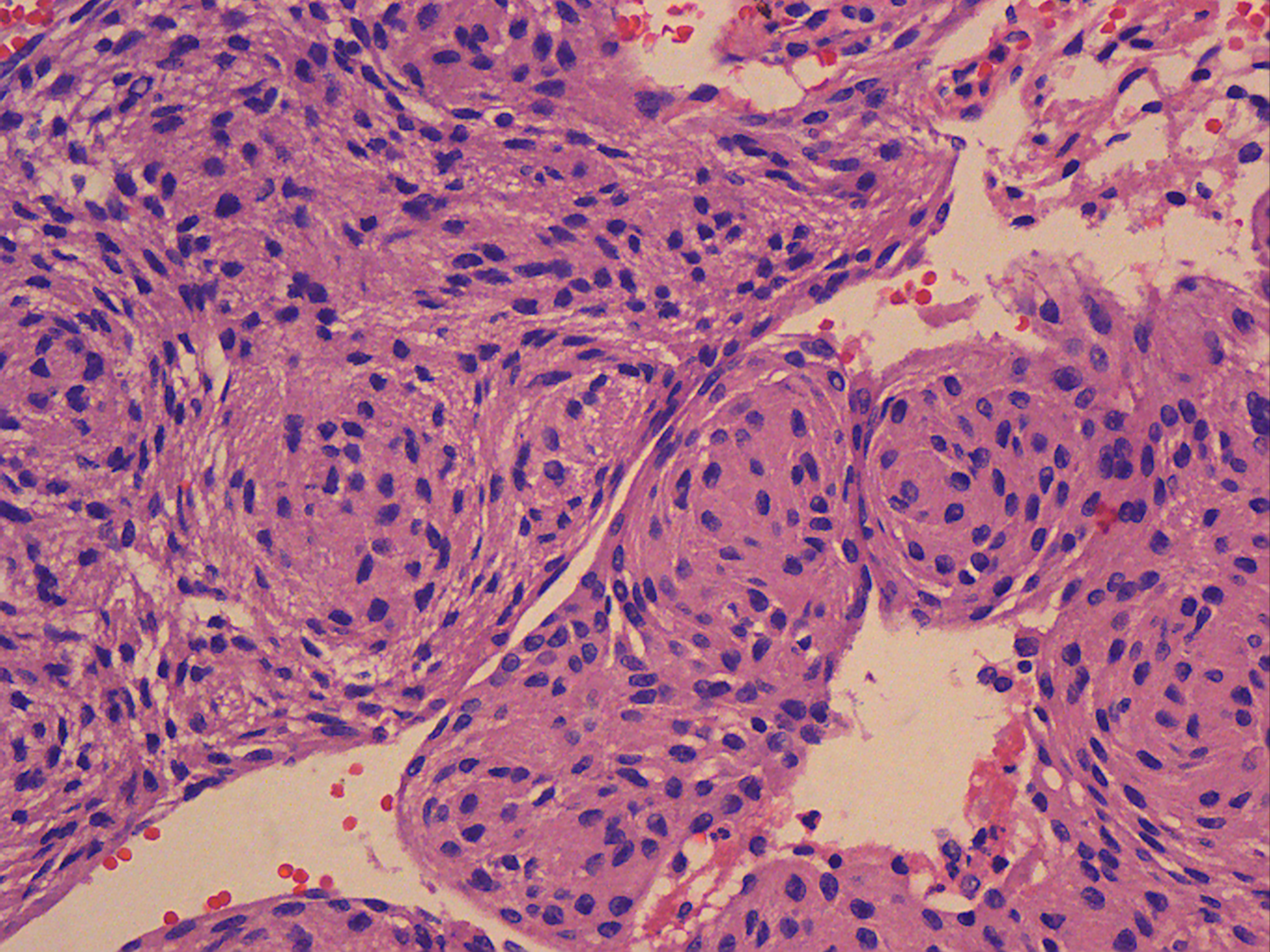

Supplement: S2 Fig — (ZIP) [file pone.0263006.s002.zip › Original Low-grade Image 1.bmp]

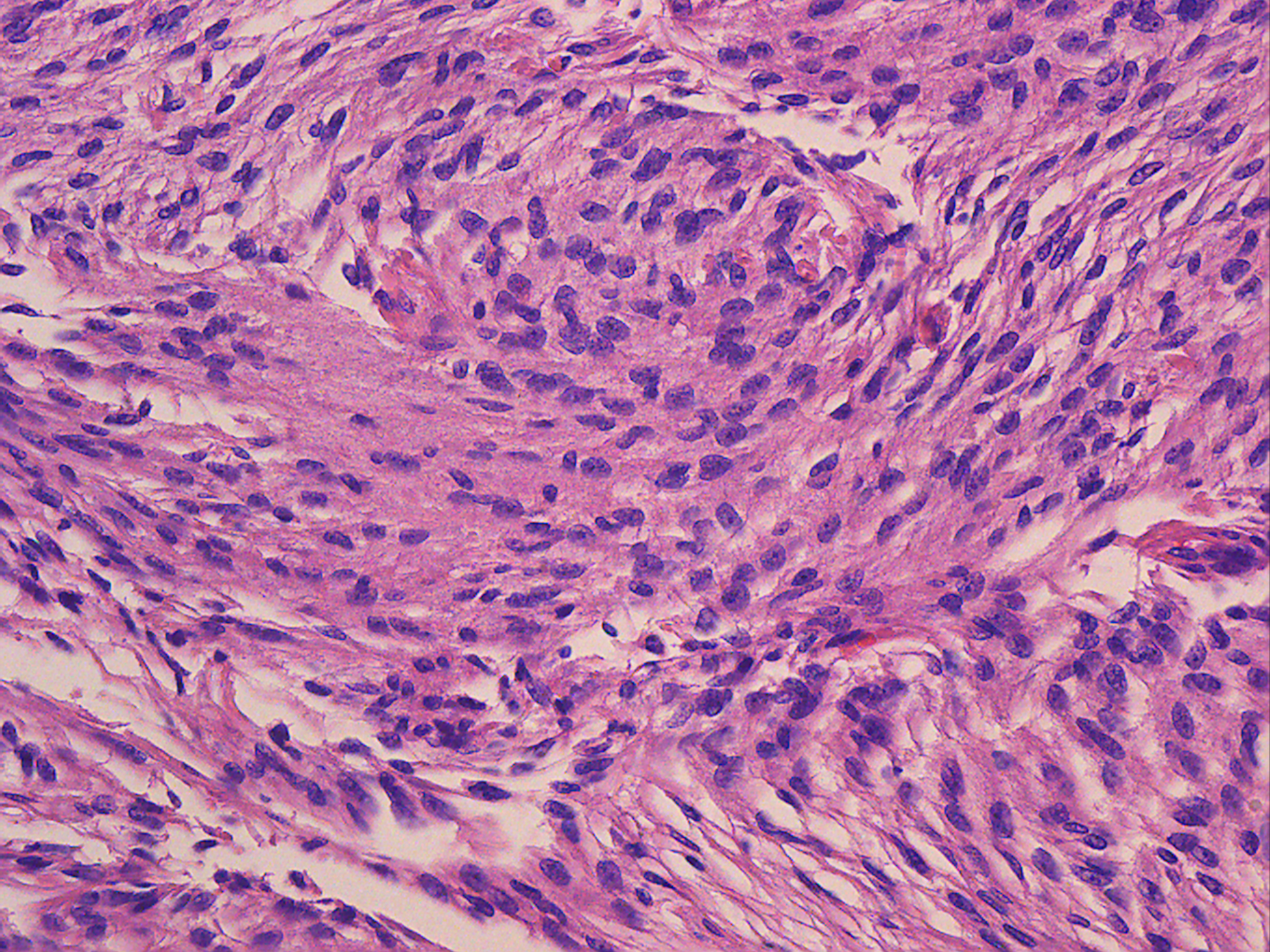

Supplement: S2 Fig — (ZIP) [file pone.0263006.s002.zip › Original Low-grade Image 2.bmp]

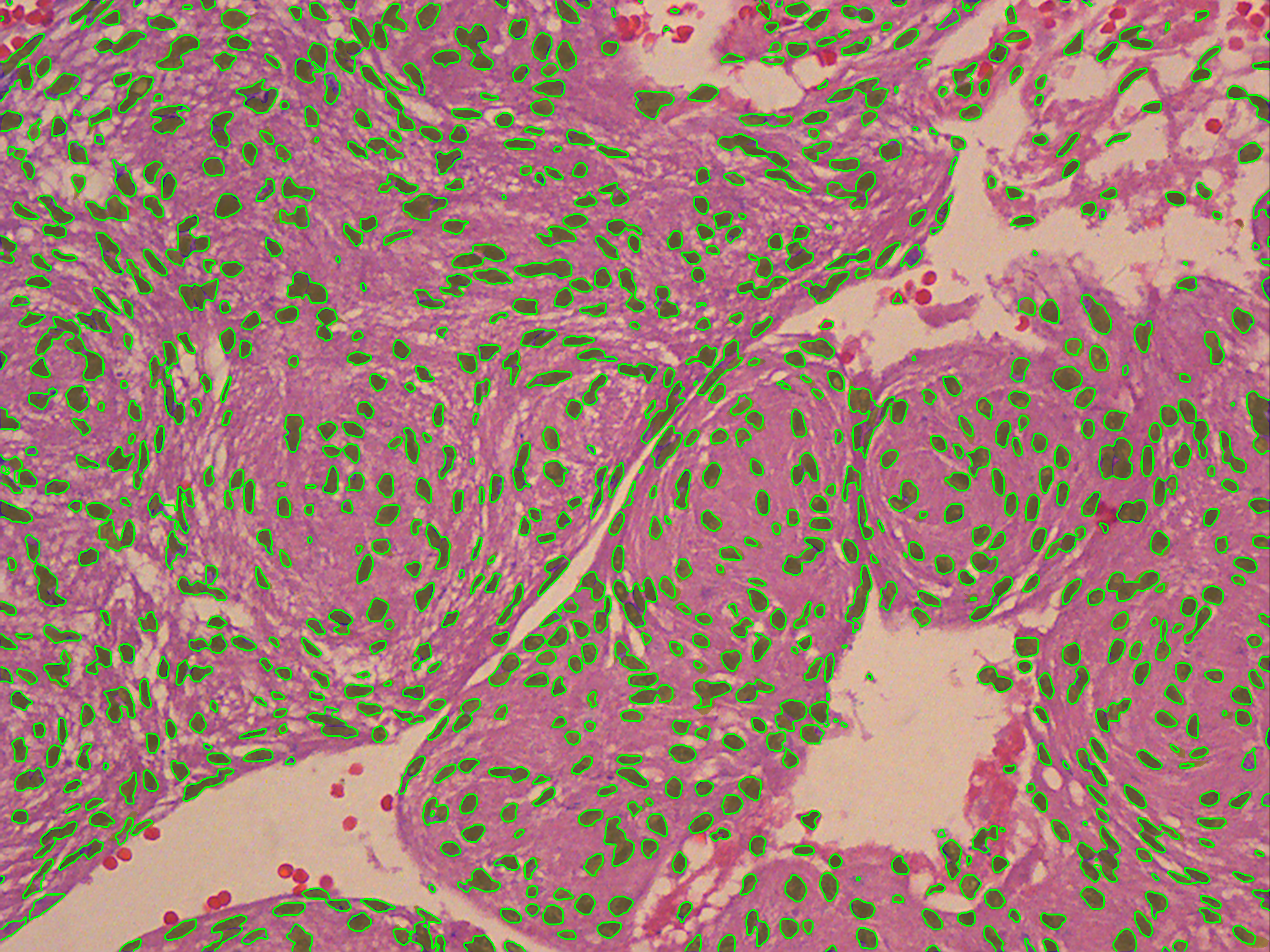

Supplement: S2 Fig — (ZIP) [file pone.0263006.s002.zip › Original Low-grade Segmentation Result 1.jpg]

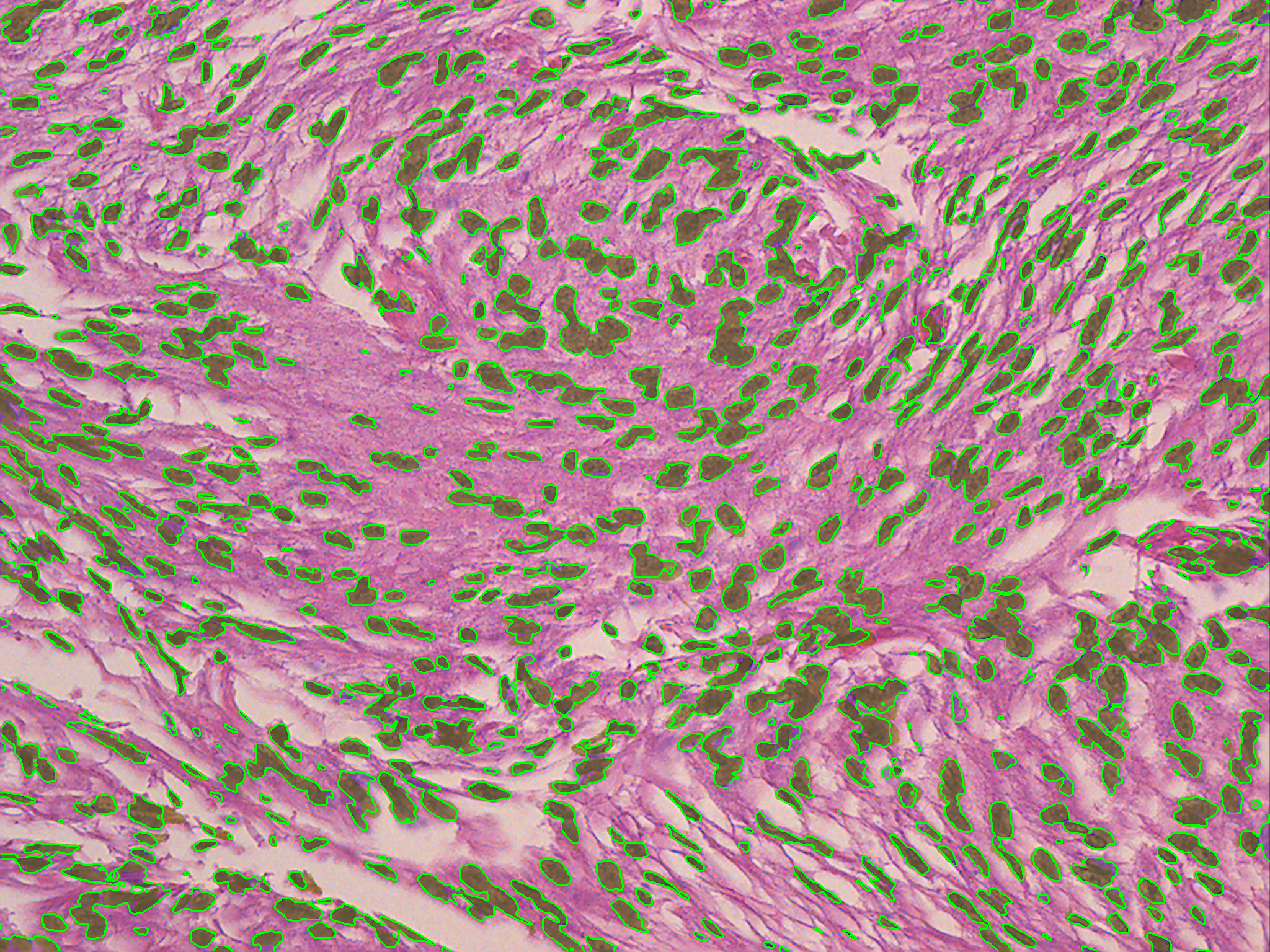

Supplement: S2 Fig — (ZIP) [file pone.0263006.s002.zip › Original Low-grade Segmentation Result 2.jpg]

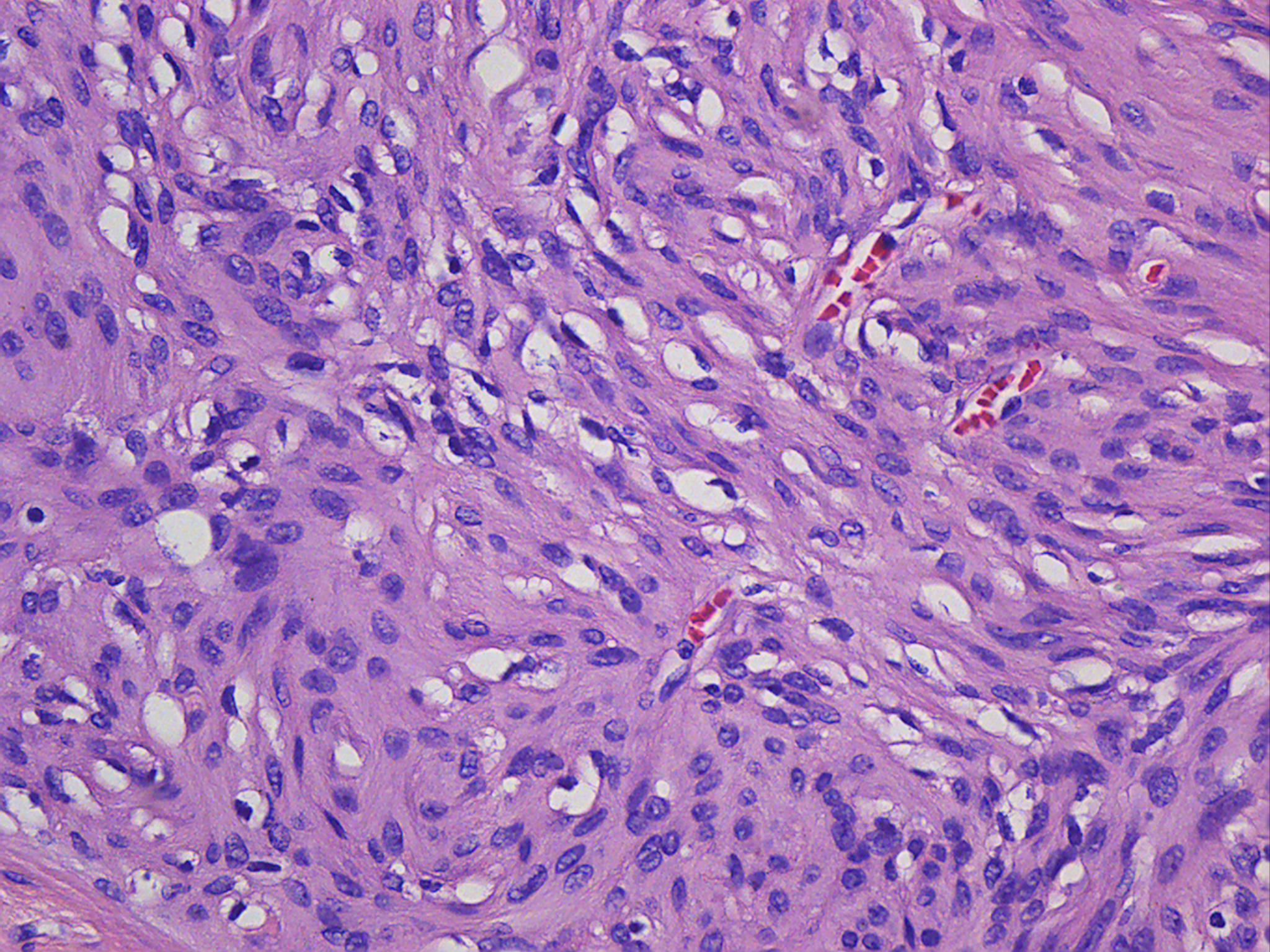

Supplement: S3 Fig — (ZIP) [file pone.0263006.s003.zip › Original HE Image 1.bmp]

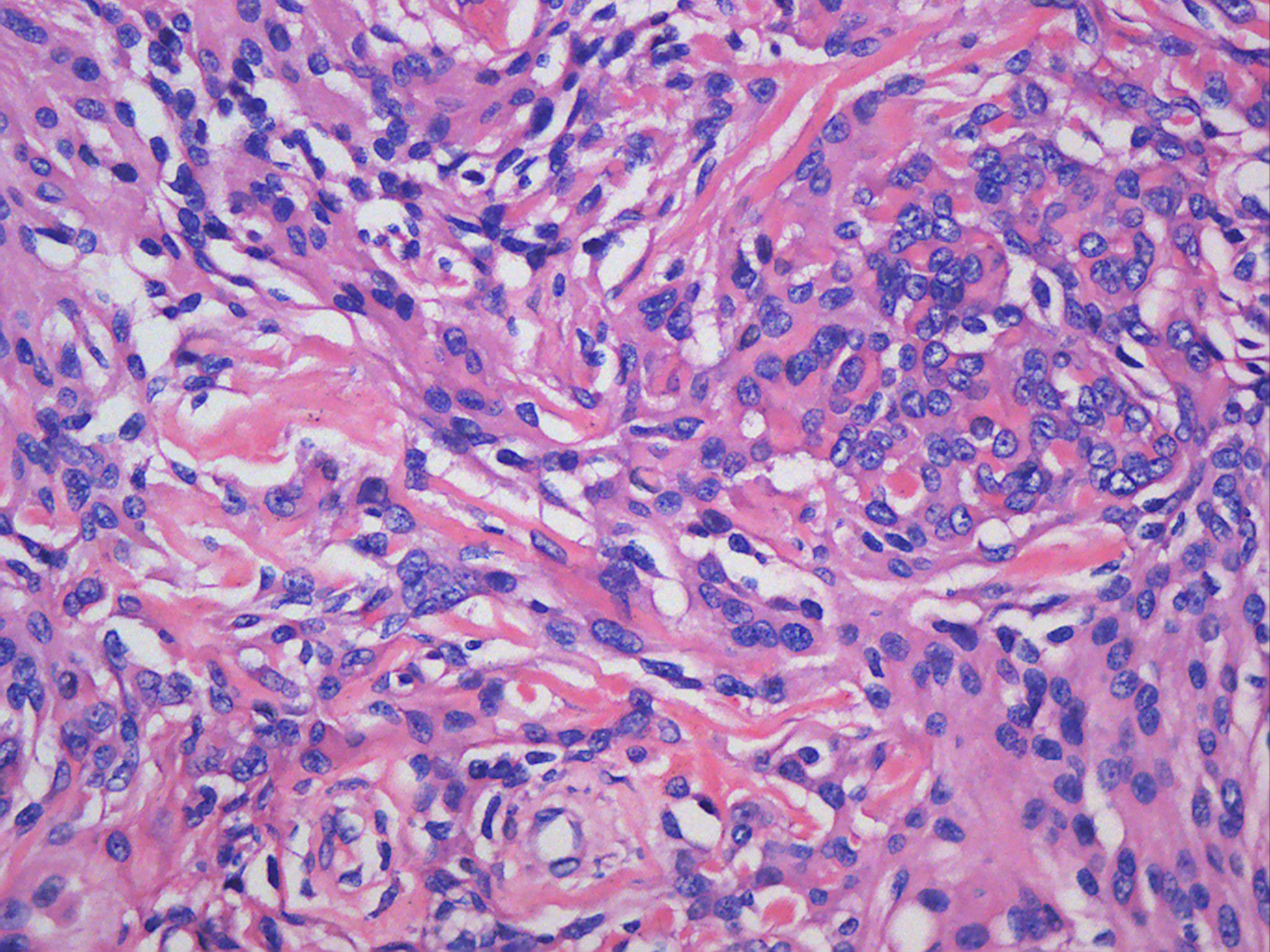

Supplement: S3 Fig — (ZIP) [file pone.0263006.s003.zip › Original HE Image 2.bmp]

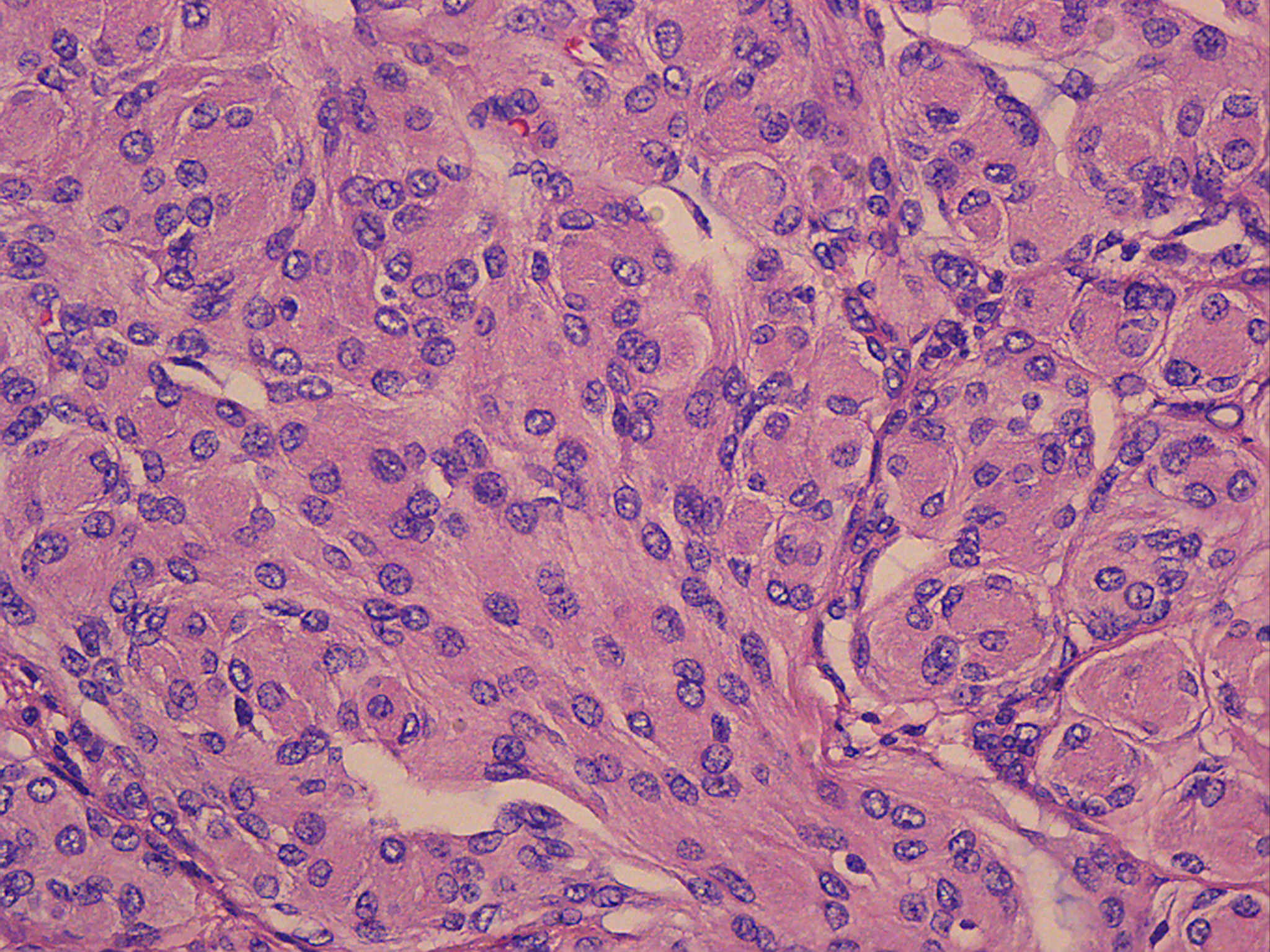

Supplement: S3 Fig — (ZIP) [file pone.0263006.s003.zip › Original HE Image 3.bmp]

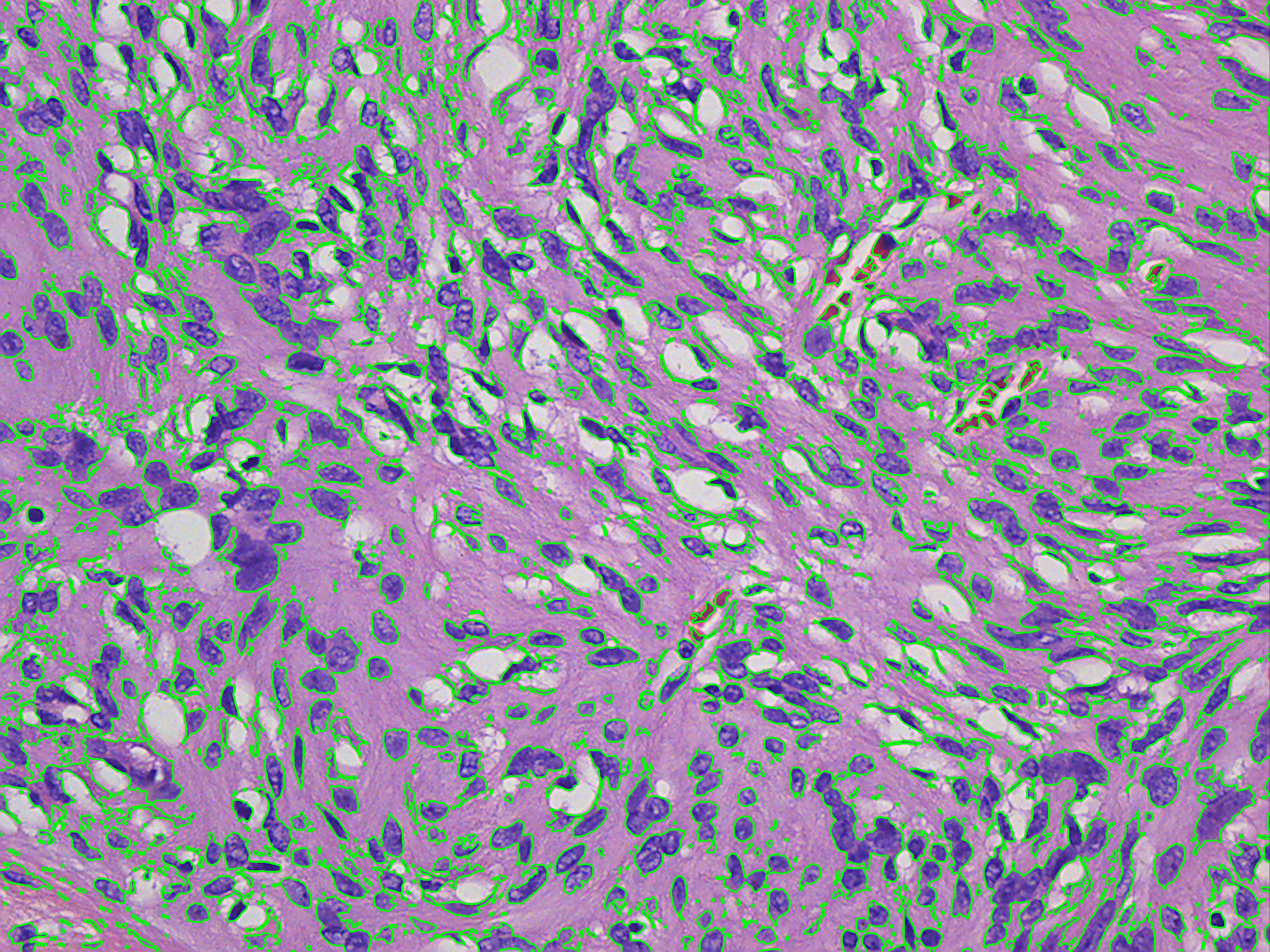

Supplement: S3 Fig — (ZIP) [file pone.0263006.s003.zip › Original K-means 1.jpg]

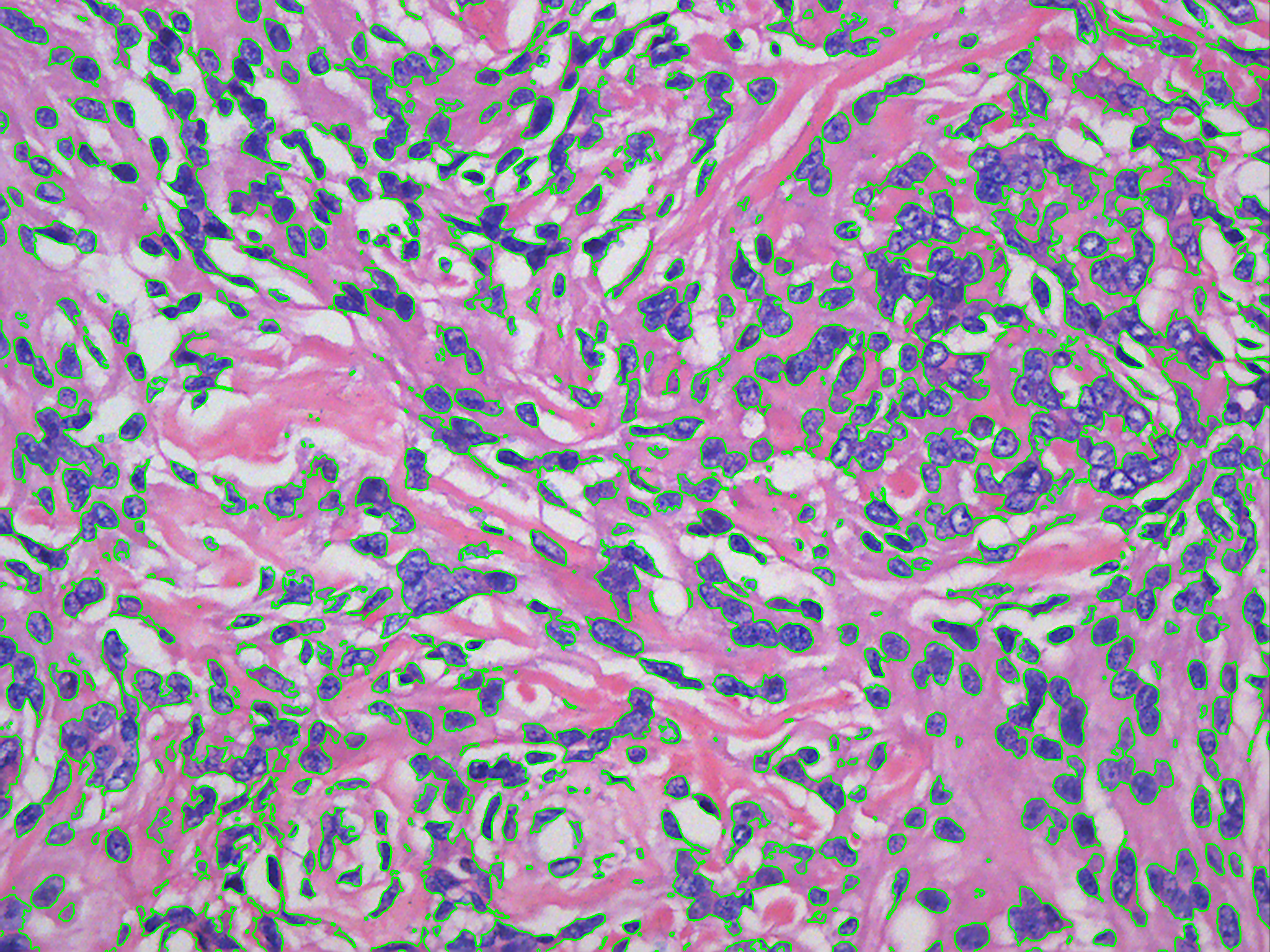

Supplement: S3 Fig — (ZIP) [file pone.0263006.s003.zip › Original K-means 2.jpg]

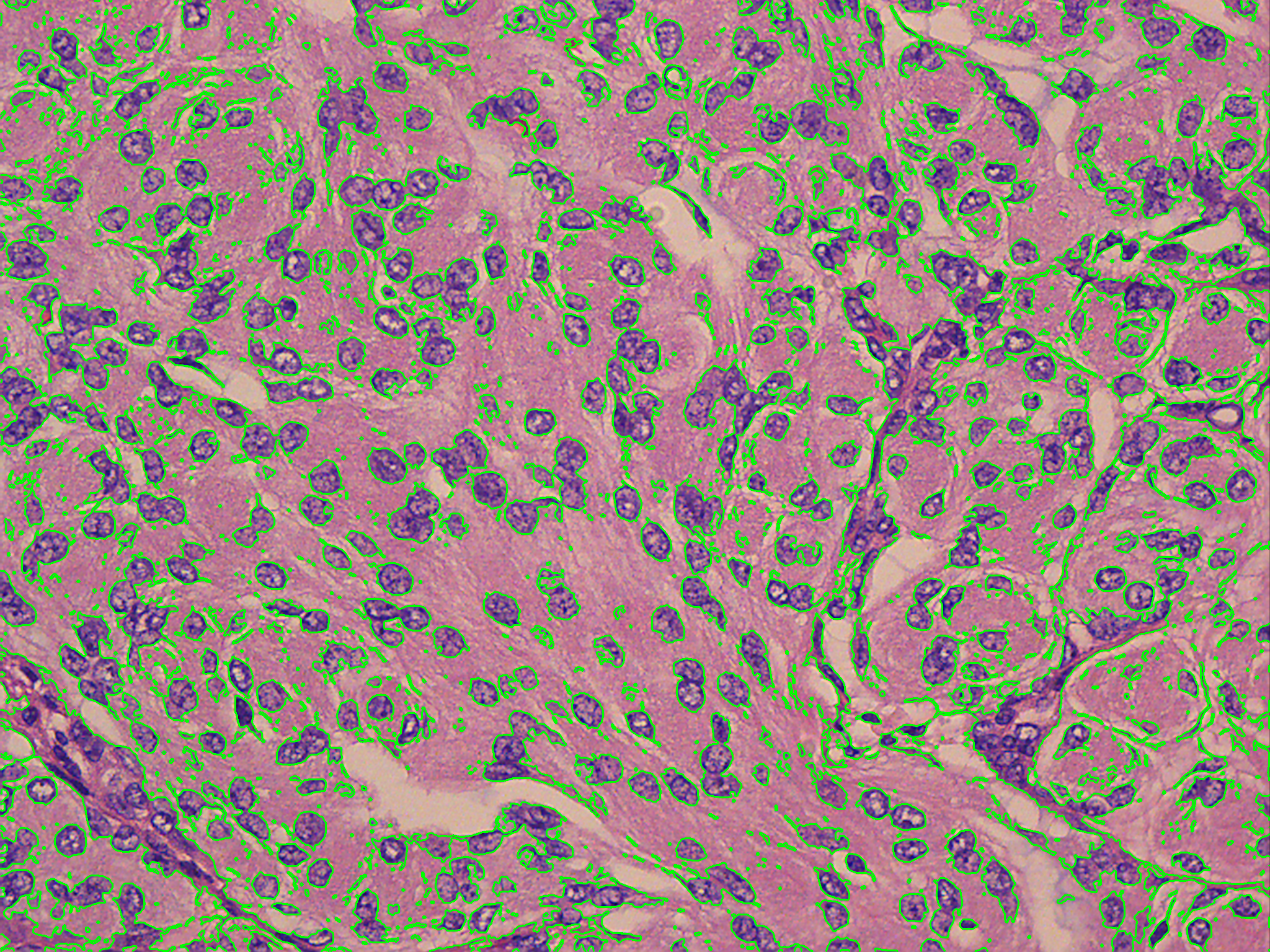

Supplement: S3 Fig — (ZIP) [file pone.0263006.s003.zip › Original K-means 3.jpg]

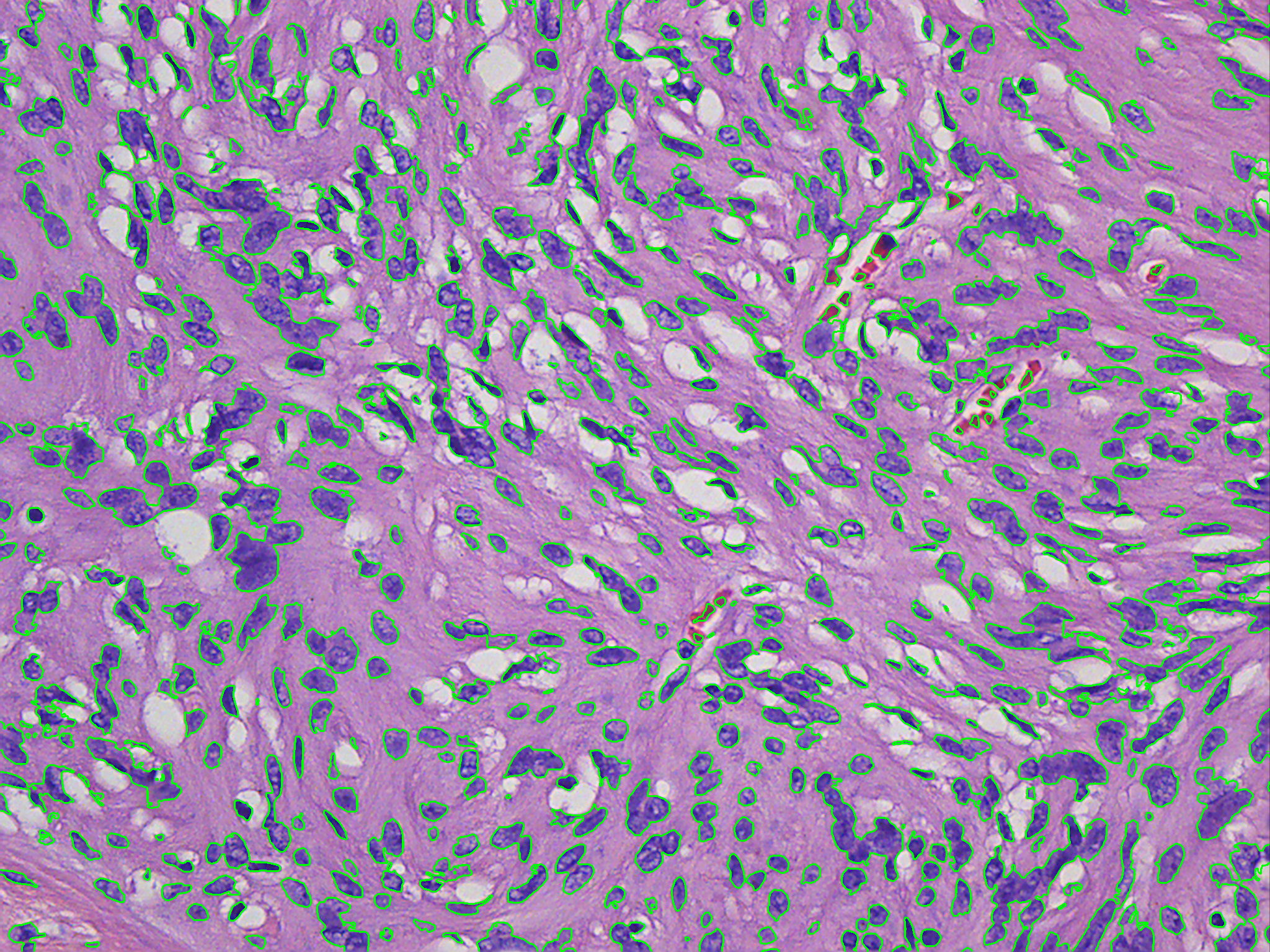

Supplement: S3 Fig — (ZIP) [file pone.0263006.s003.zip › Original Ours 1.jpg]

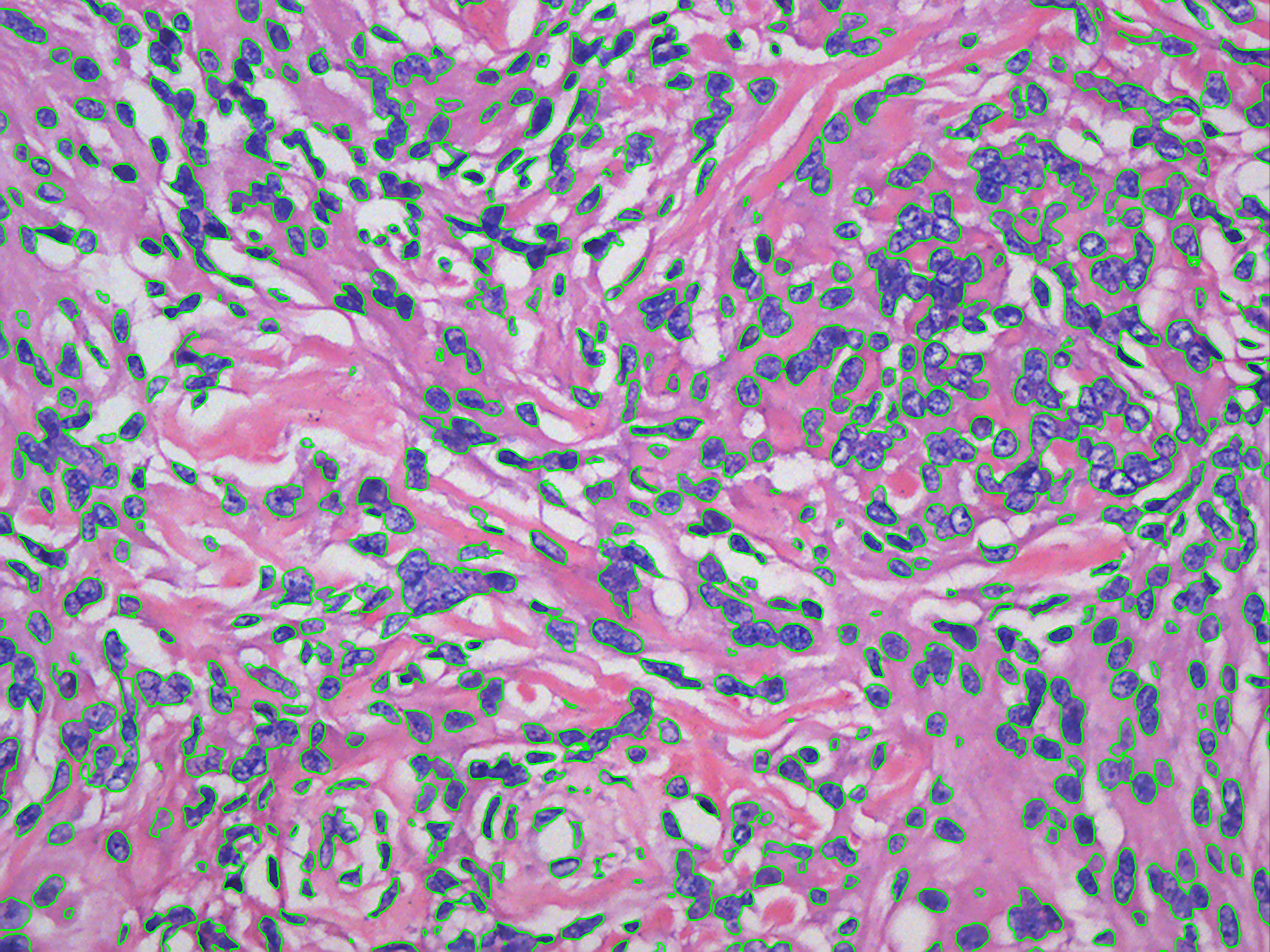

Supplement: S3 Fig — (ZIP) [file pone.0263006.s003.zip › Original Ours 2.jpg]

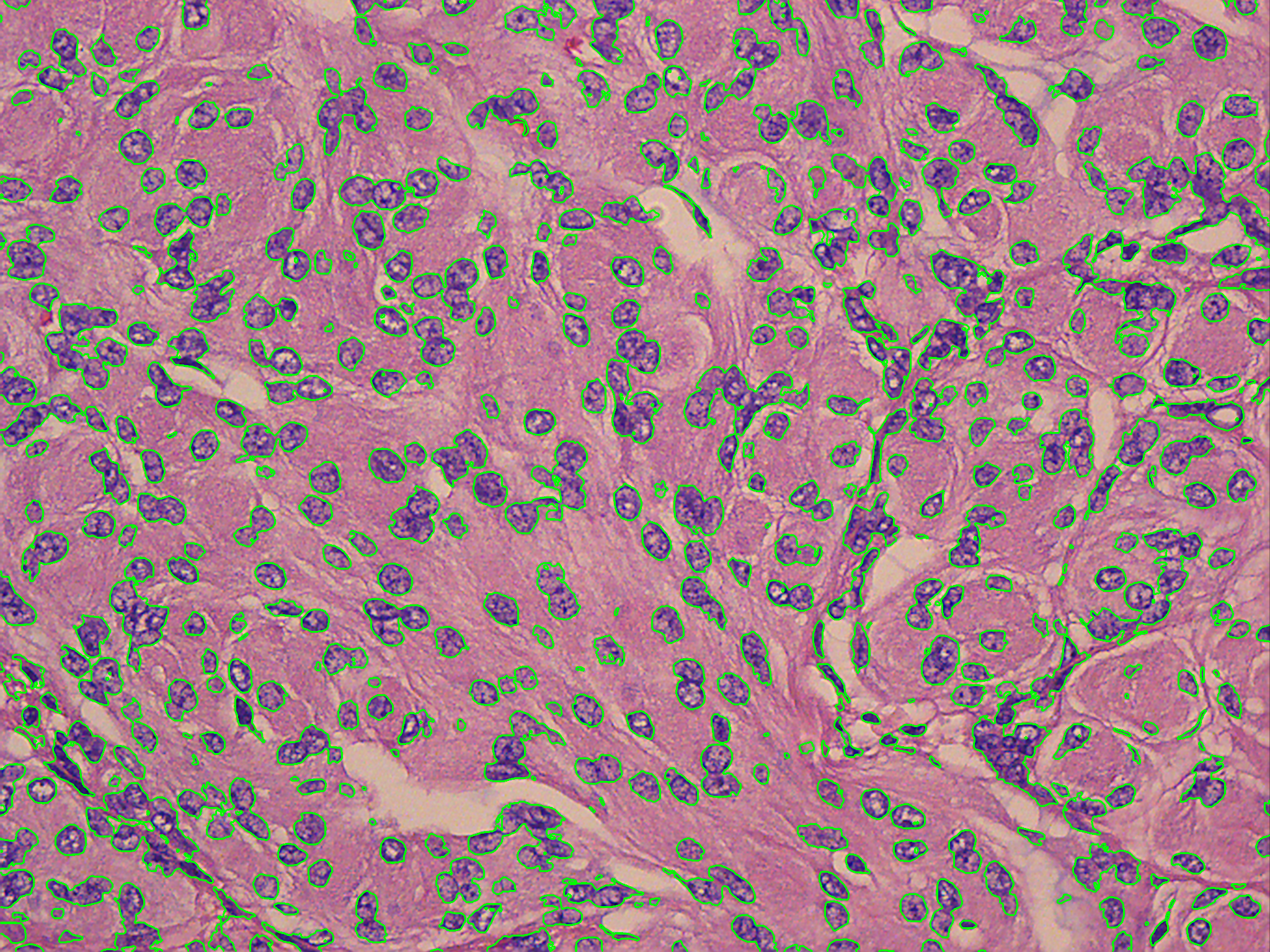

Supplement: S3 Fig — (ZIP) [file pone.0263006.s003.zip › Original Ours 3.jpg]

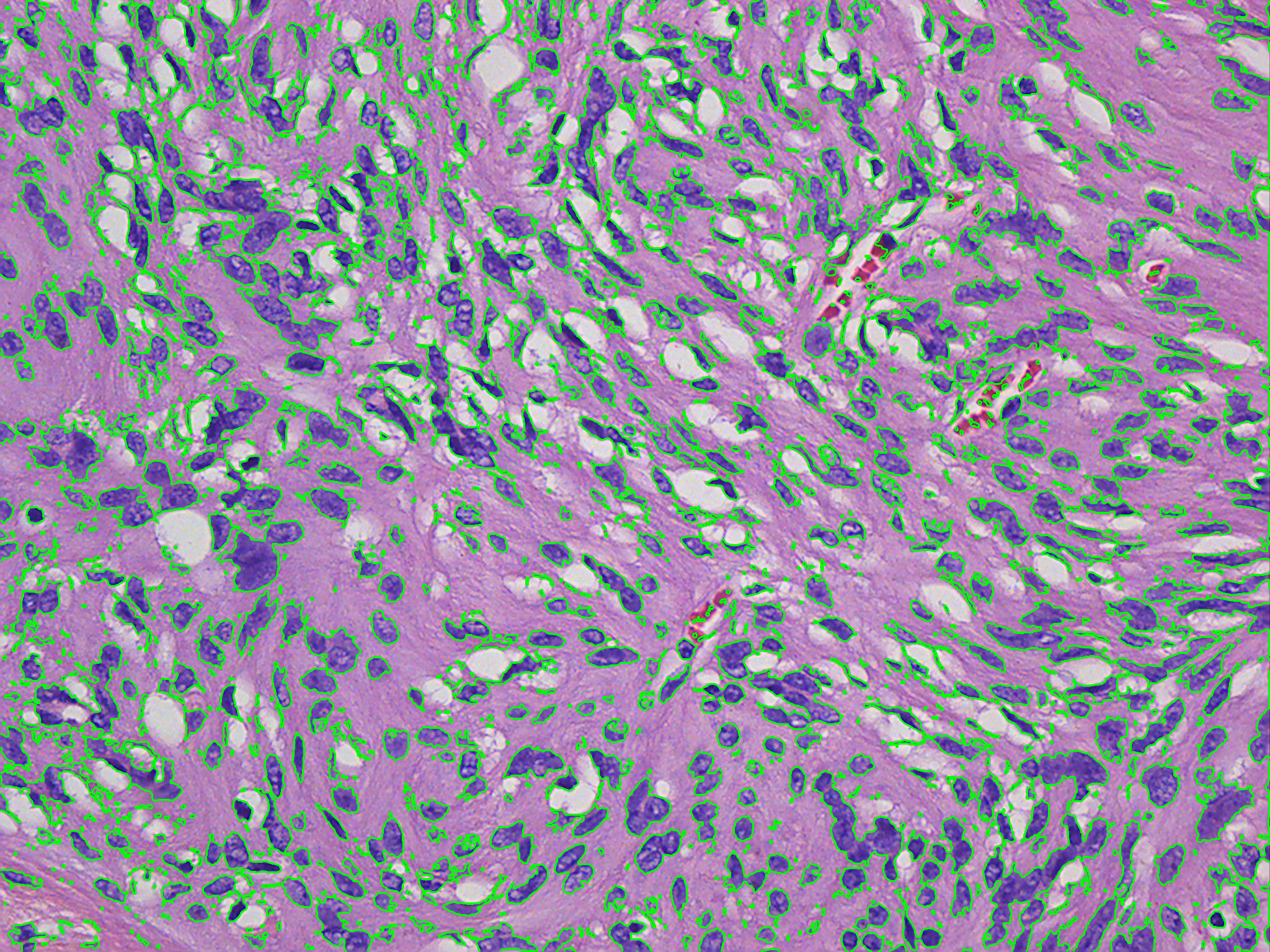

Supplement: S3 Fig — (ZIP) [file pone.0263006.s003.zip › Original SVM 1.jpg]

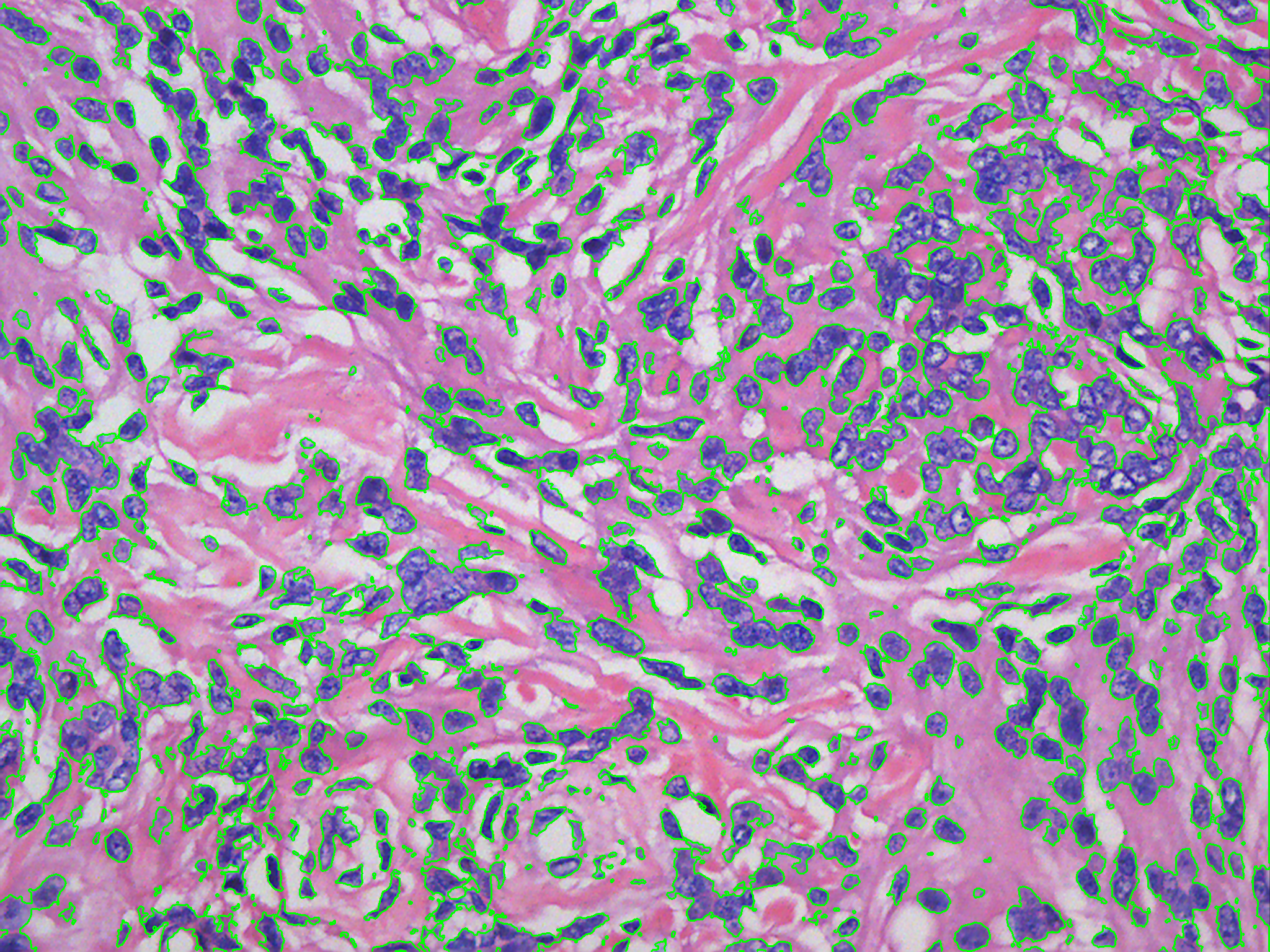

Supplement: S3 Fig — (ZIP) [file pone.0263006.s003.zip › Original SVM 2.jpg]

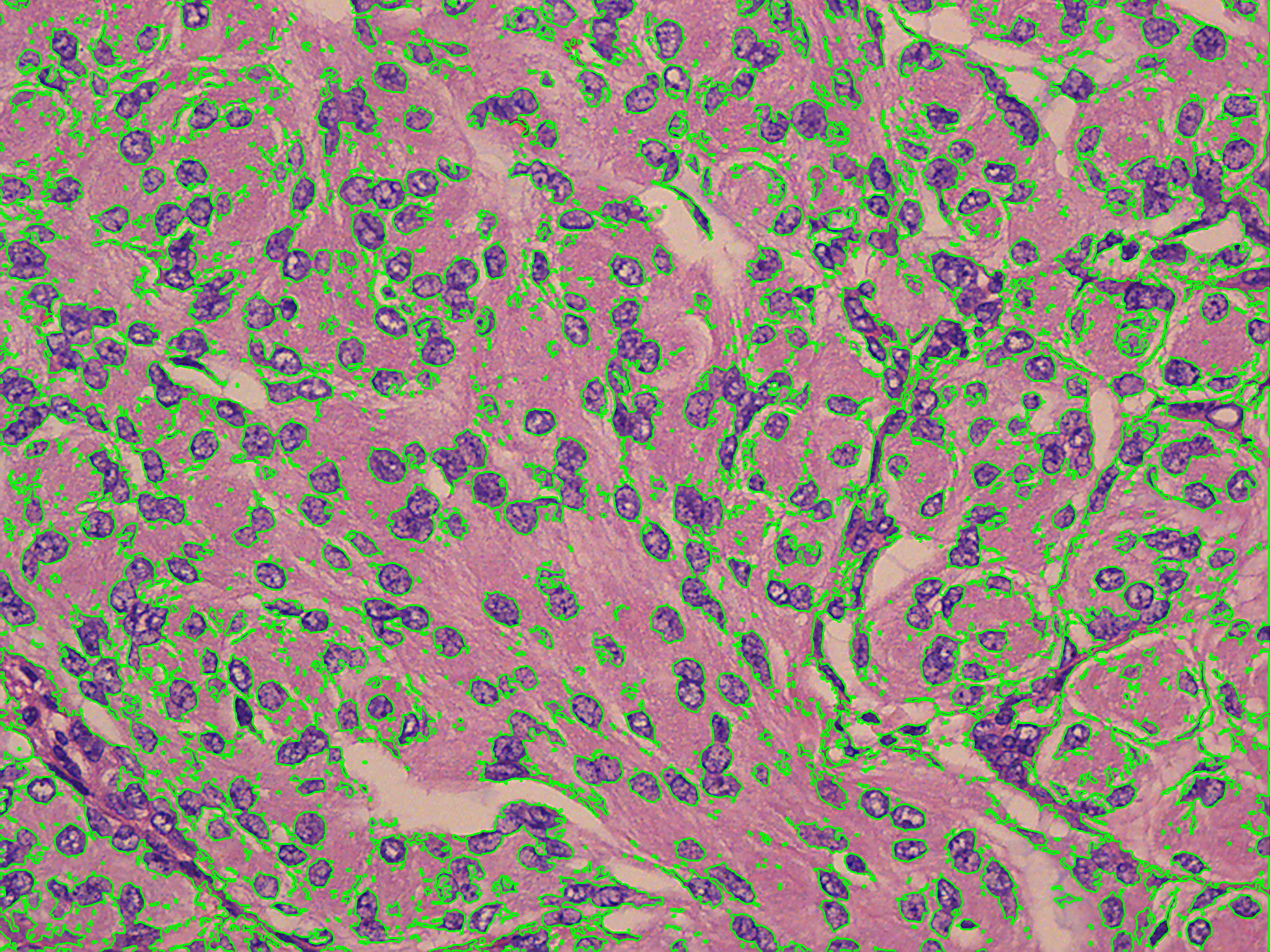

Supplement: S3 Fig — (ZIP) [file pone.0263006.s003.zip › Original SVM 3.jpg]

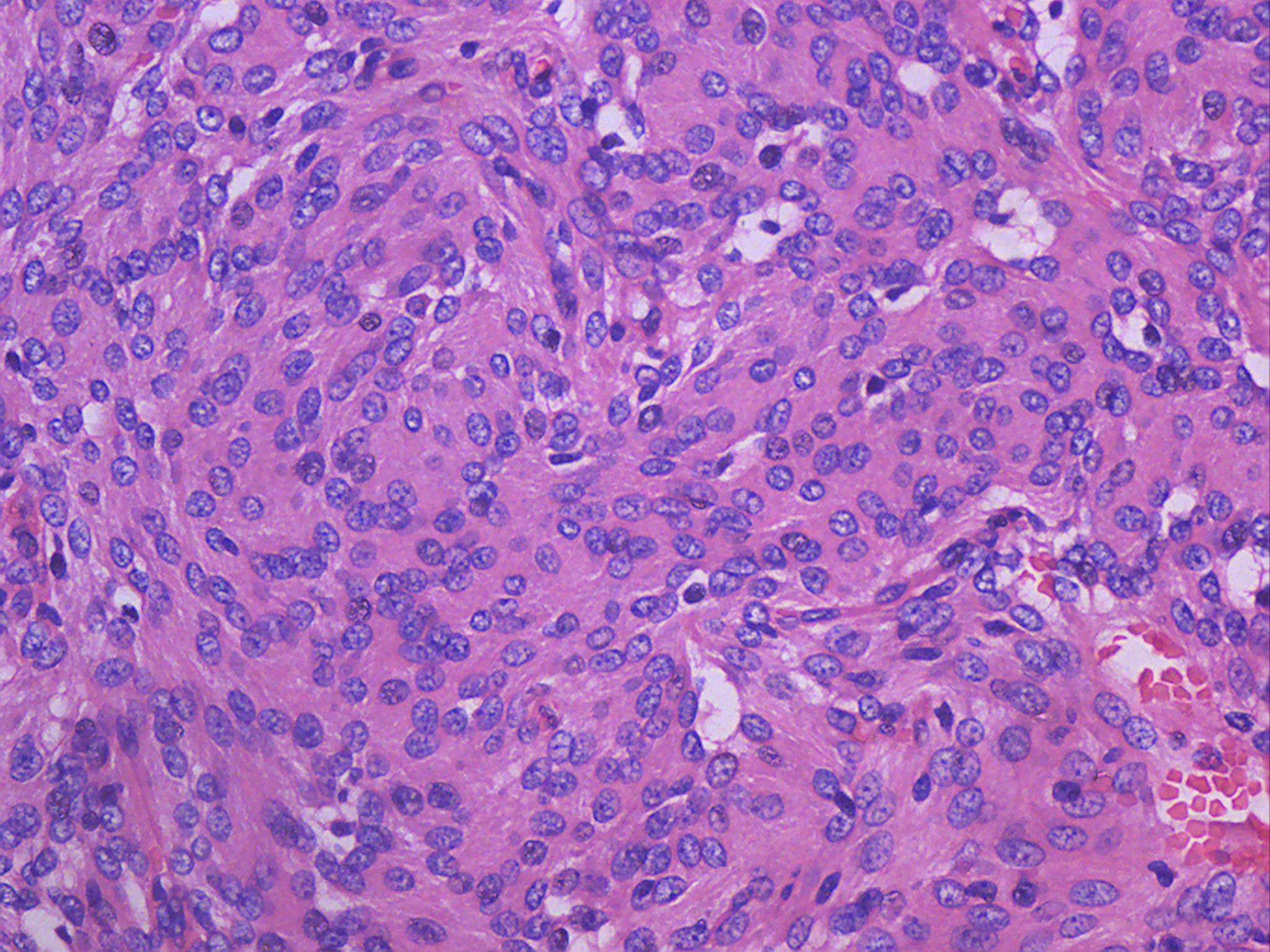

Supplement: S4 Fig — (ZIP) [file pone.0263006.s004.zip › Original HE Image 3.tif]

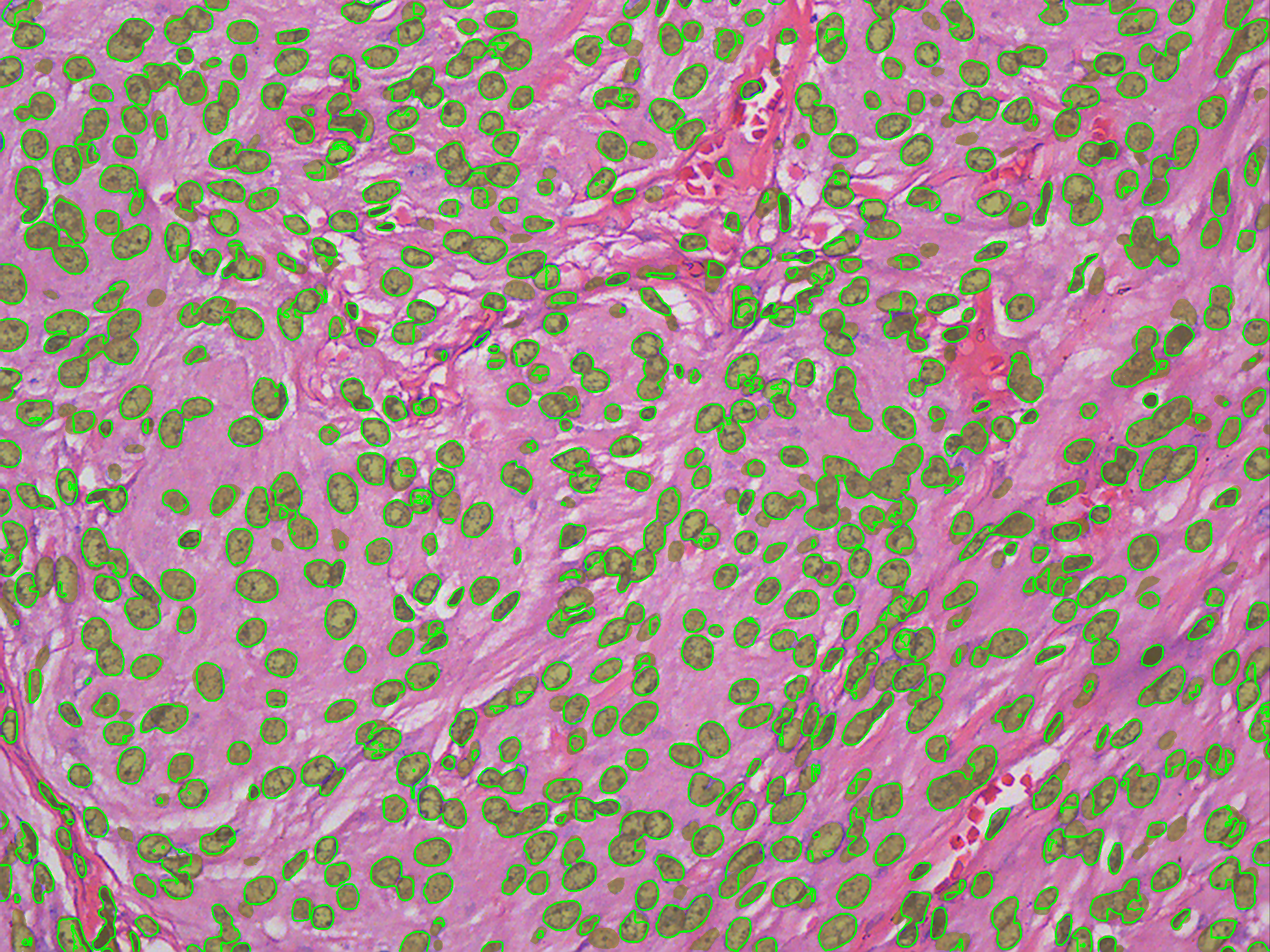

Supplement: S5 Fig — (ZIP) [file pone.0263006.s005.zip › Original AttU-net 1.jpg]

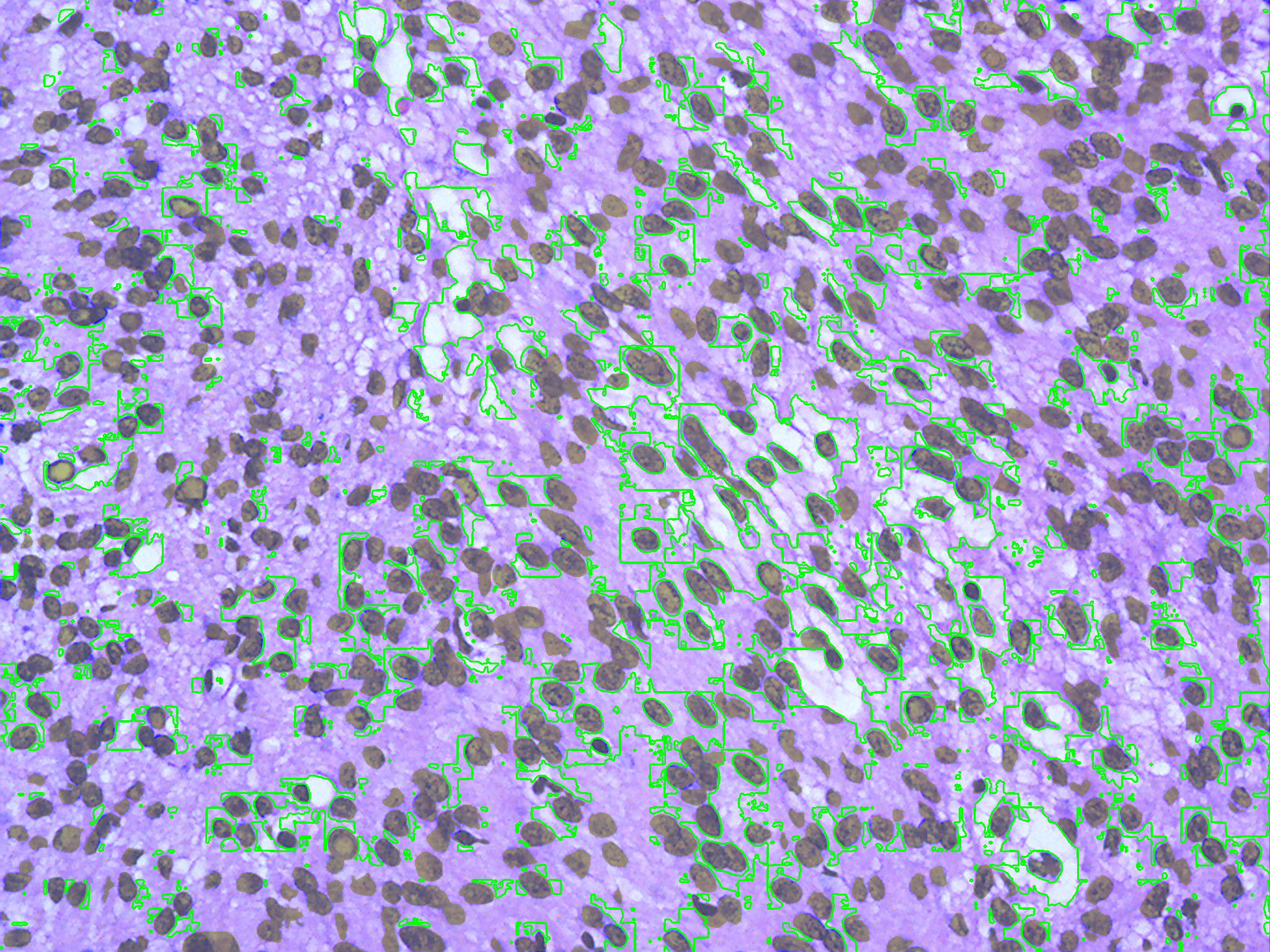

Supplement: S5 Fig — (ZIP) [file pone.0263006.s005.zip › Original AttU-net 2.jpg]

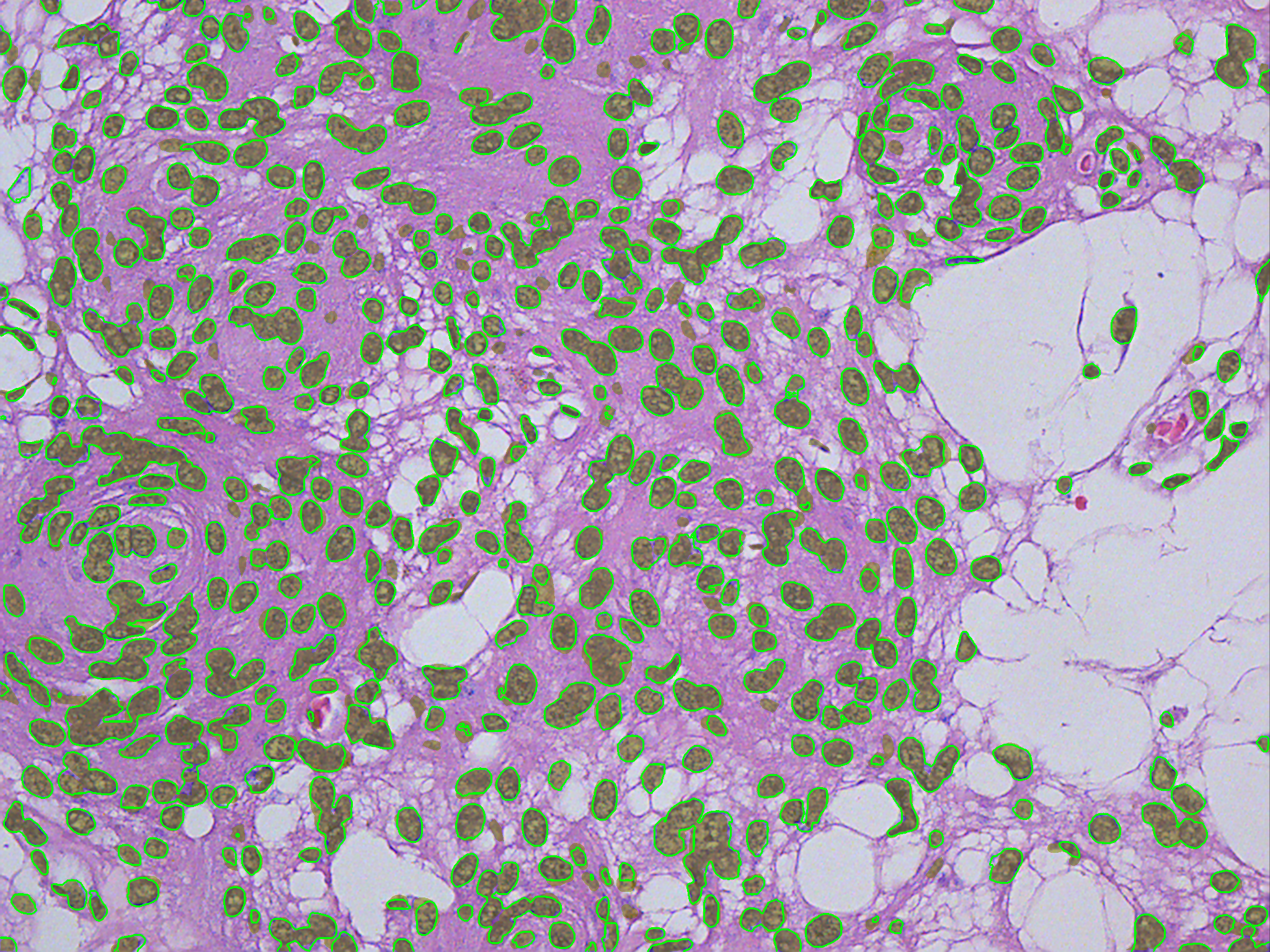

Supplement: S5 Fig — (ZIP) [file pone.0263006.s005.zip › Original AttU-net 3.jpg]

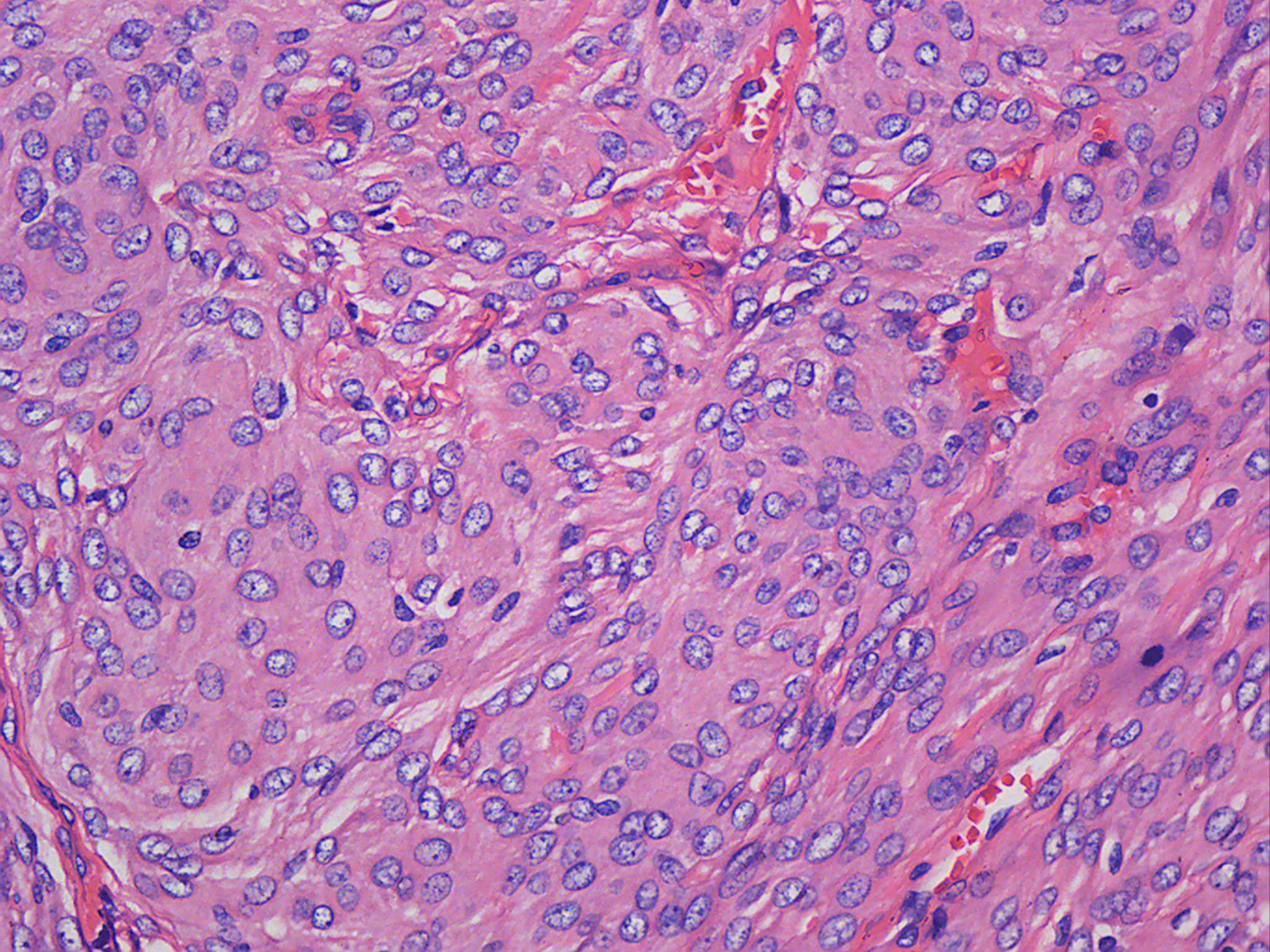

Supplement: S5 Fig — (ZIP) [file pone.0263006.s005.zip › Original HE Image 1.bmp]

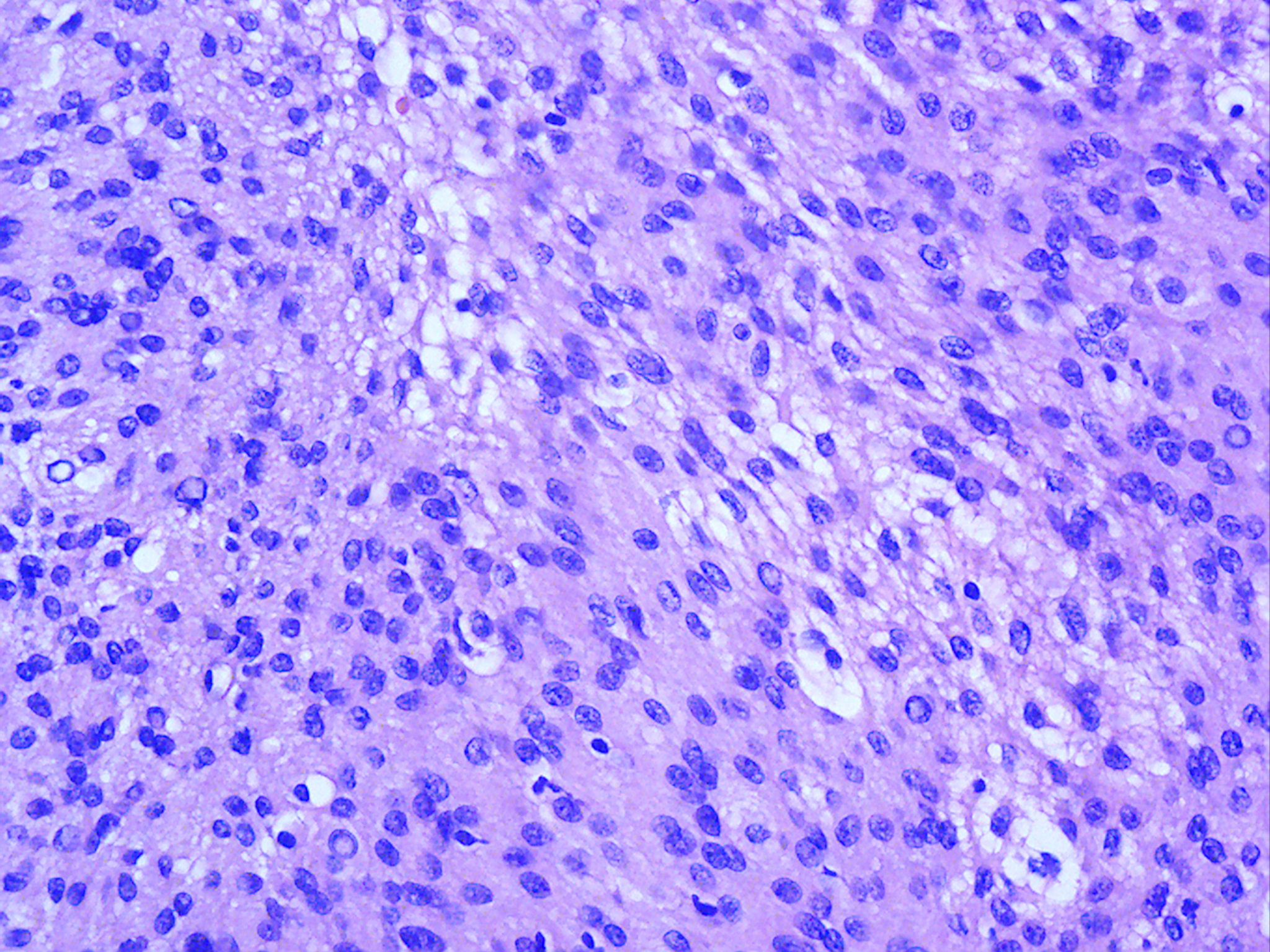

Supplement: S5 Fig — (ZIP) [file pone.0263006.s005.zip › Original HE Image 2.bmp]

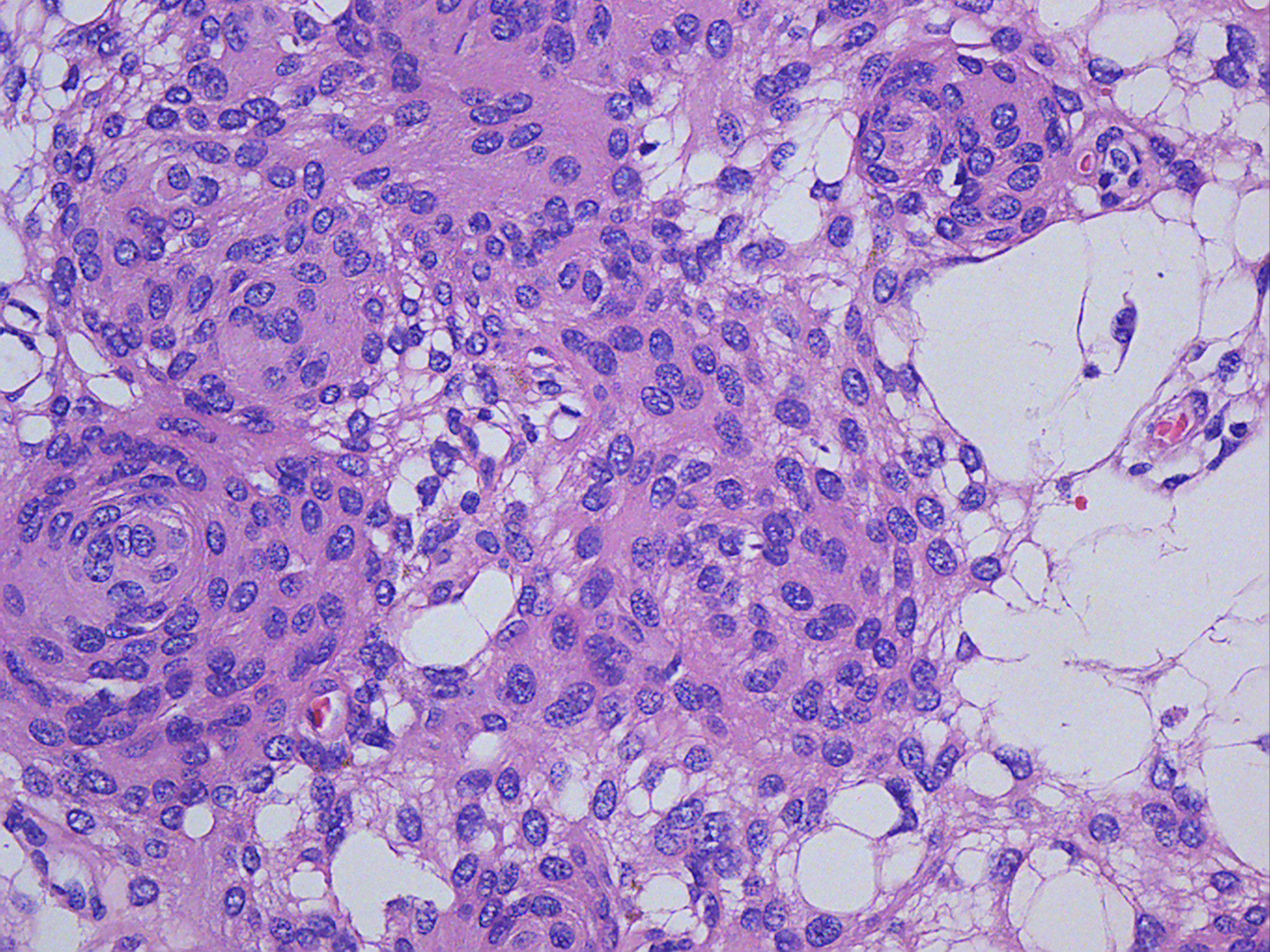

Supplement: S5 Fig — (ZIP) [file pone.0263006.s005.zip › Original HE Image 3.bmp]

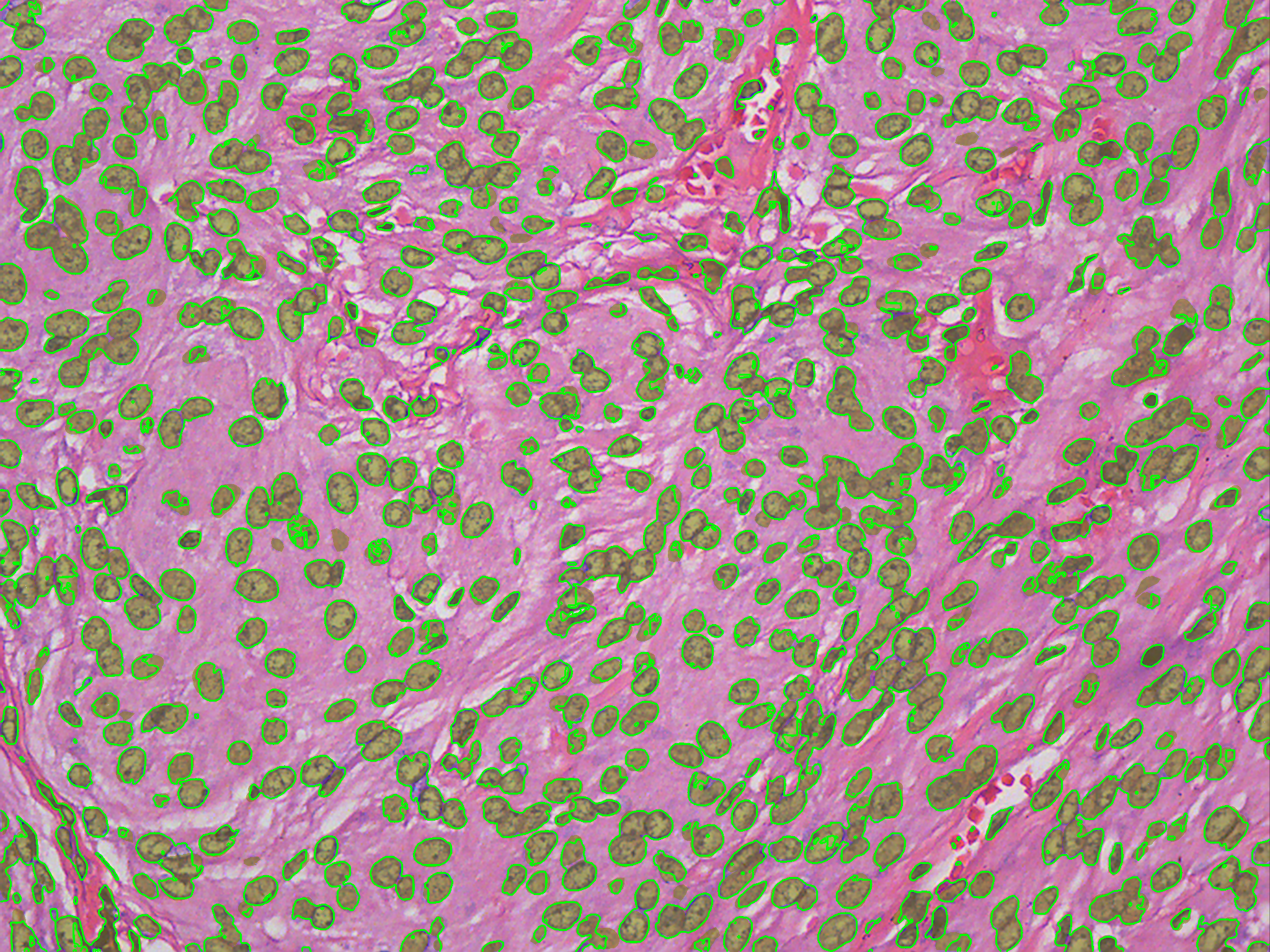

Supplement: S5 Fig — (ZIP) [file pone.0263006.s005.zip › Original Ours 1.jpg]

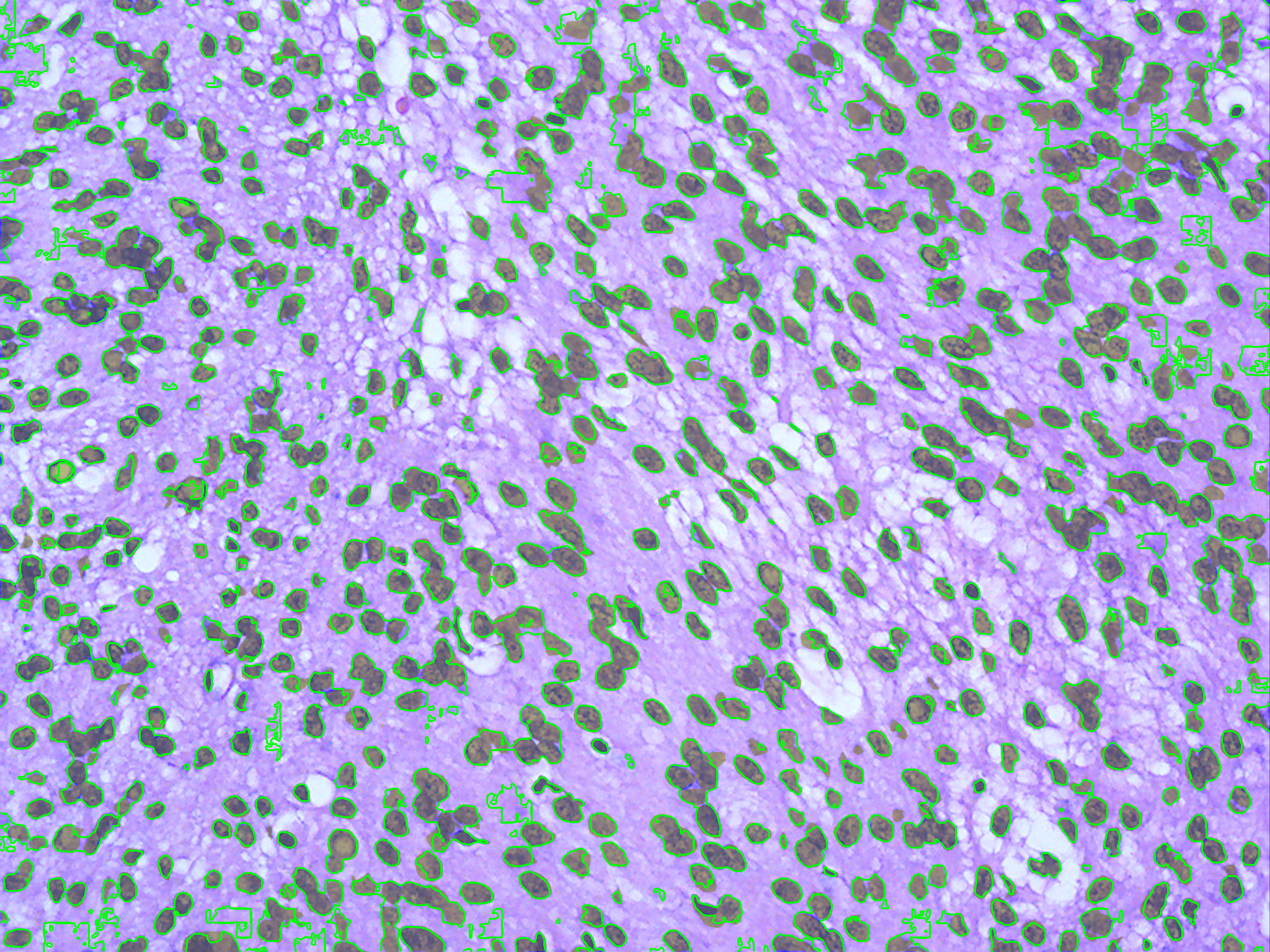

Supplement: S5 Fig — (ZIP) [file pone.0263006.s005.zip › Original Ours 2.jpg]

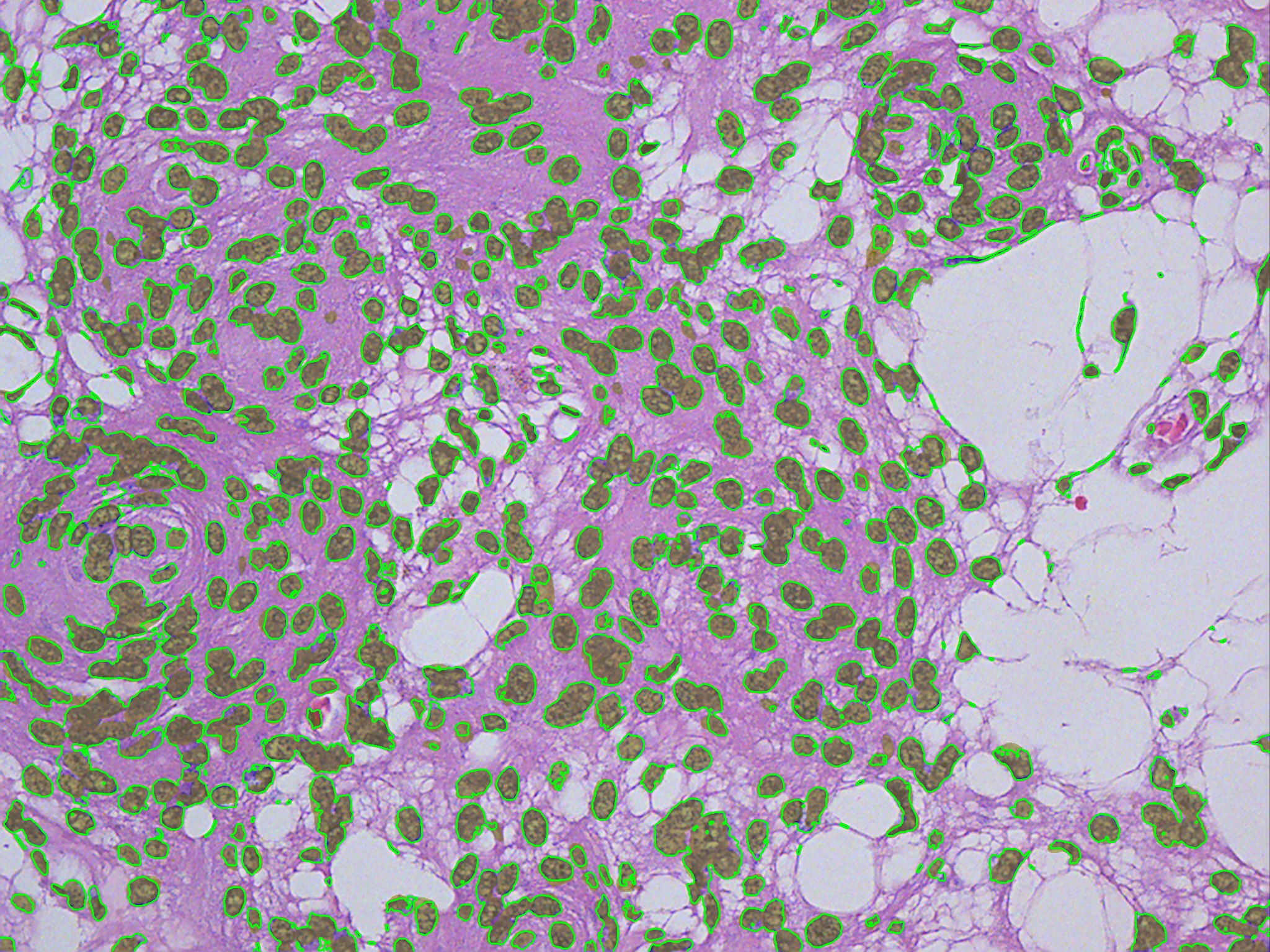

Supplement: S5 Fig — (ZIP) [file pone.0263006.s005.zip › Original Ours 3.jpg]

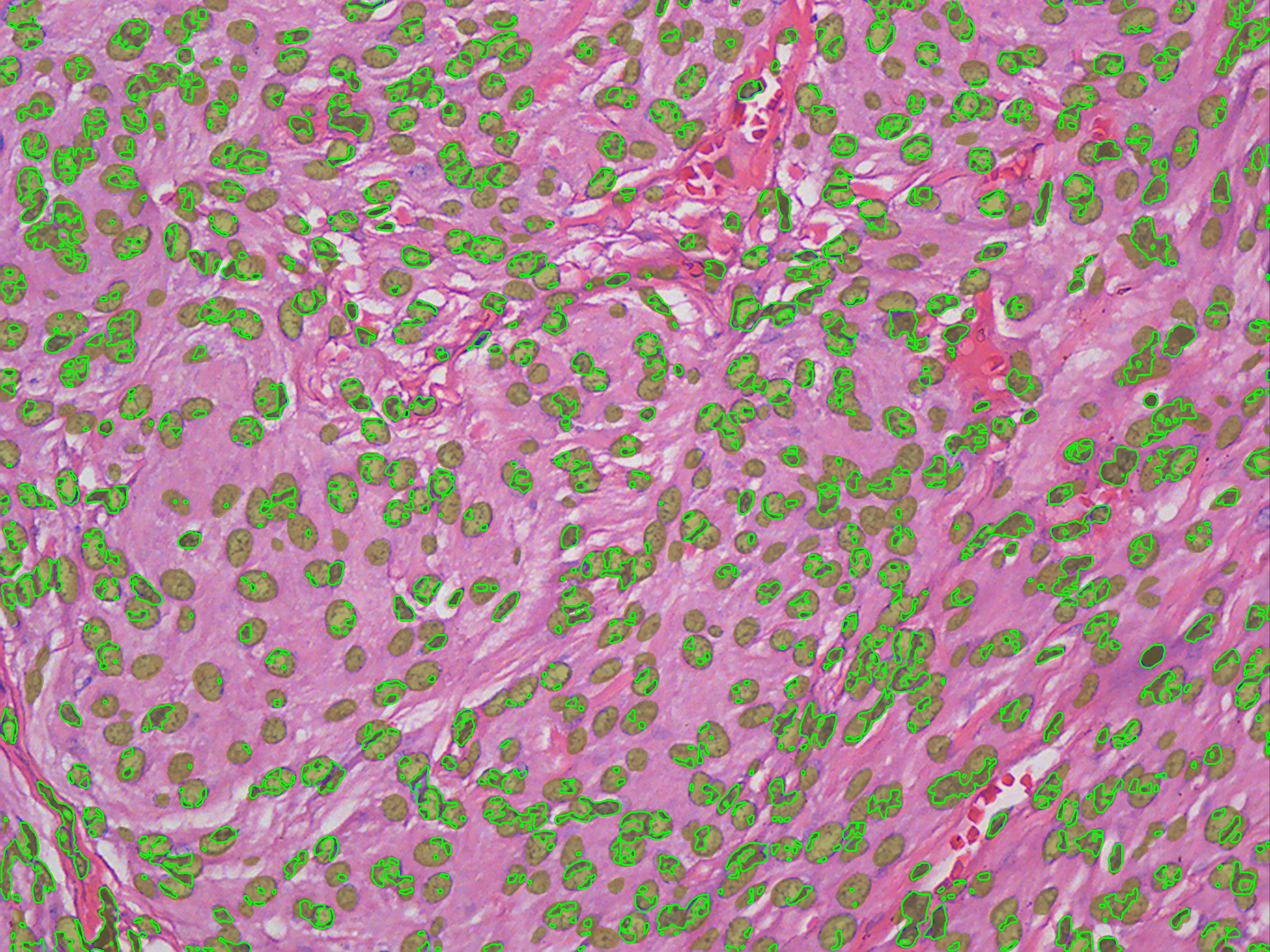

Supplement: S6 Fig — (ZIP) [file pone.0263006.s006.zip › Original R2U-net 1.jpg]

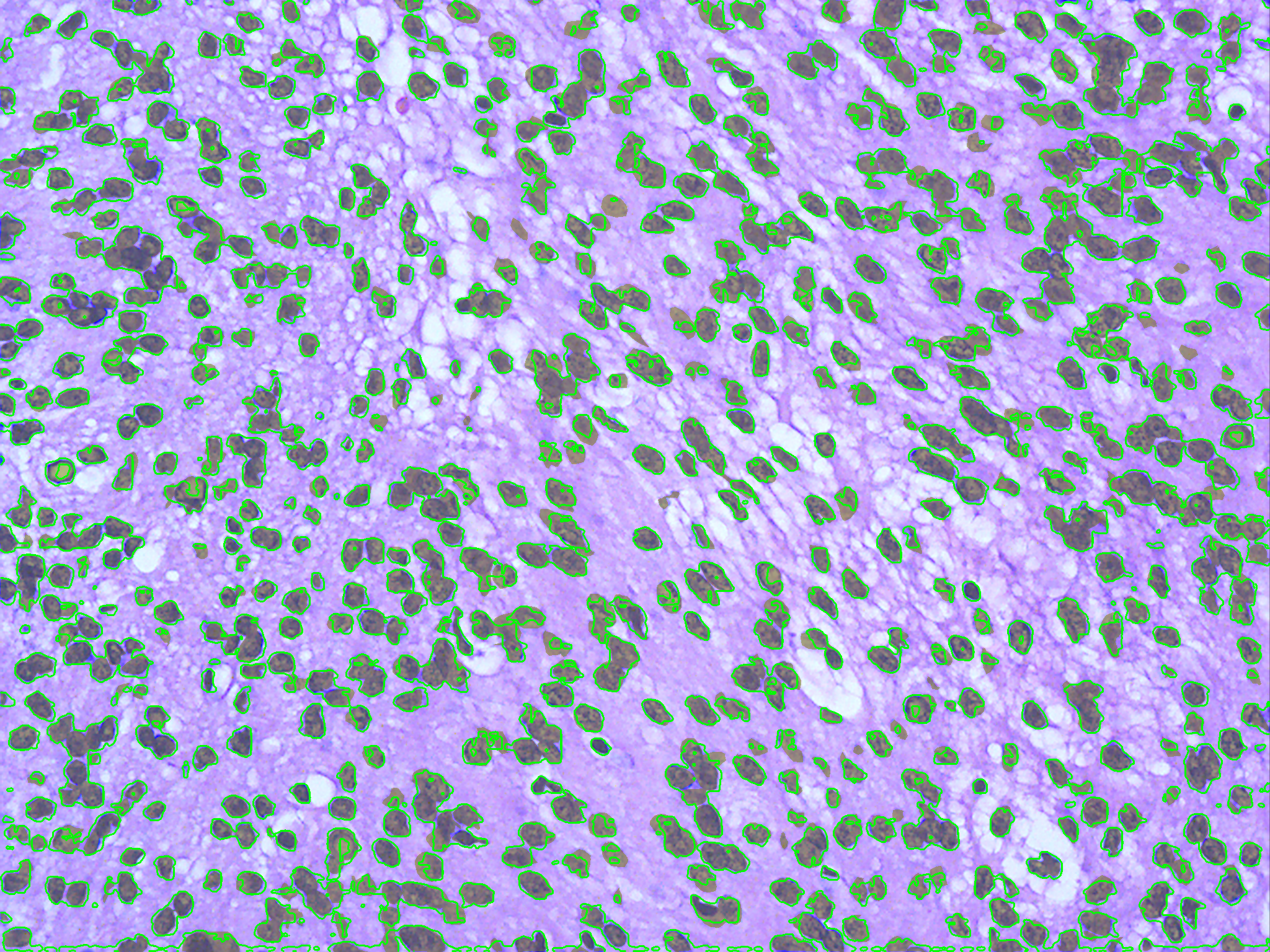

Supplement: S6 Fig — (ZIP) [file pone.0263006.s006.zip › Original R2U-net 2.jpg]

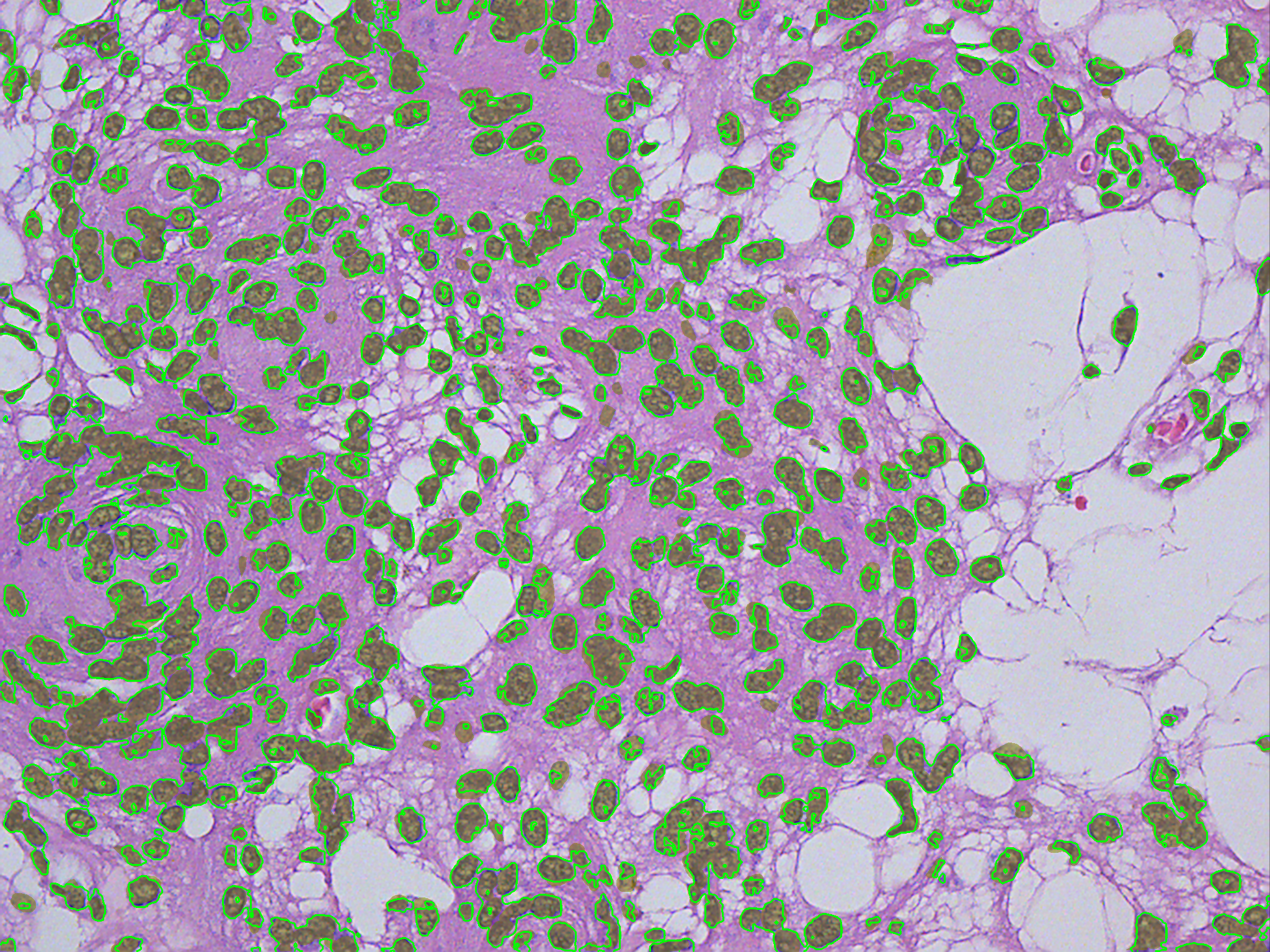

Supplement: S6 Fig — (ZIP) [file pone.0263006.s006.zip › Original R2U-net 3.jpg]

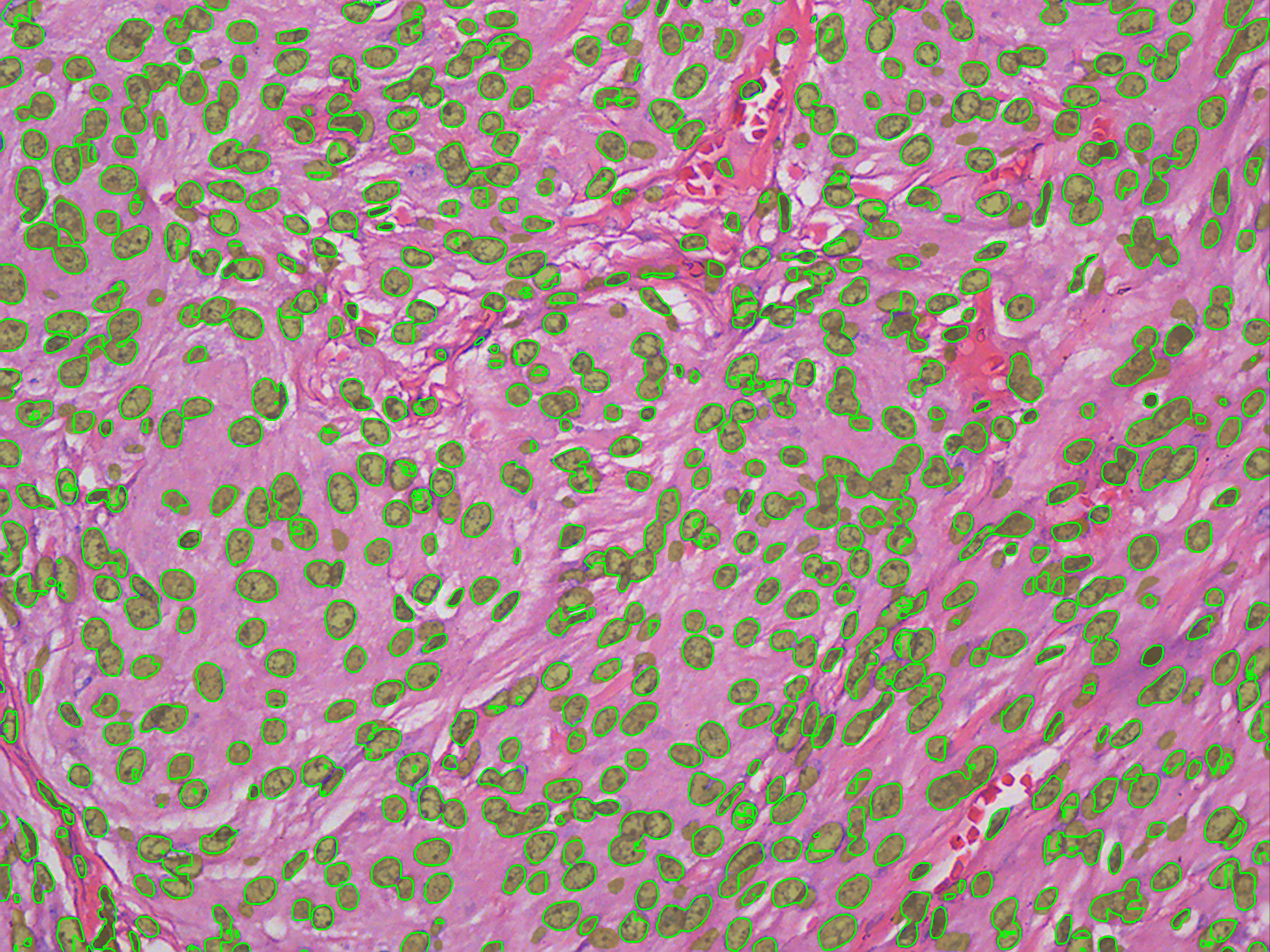

Supplement: S6 Fig — (ZIP) [file pone.0263006.s006.zip › Original U-net 1.jpg]

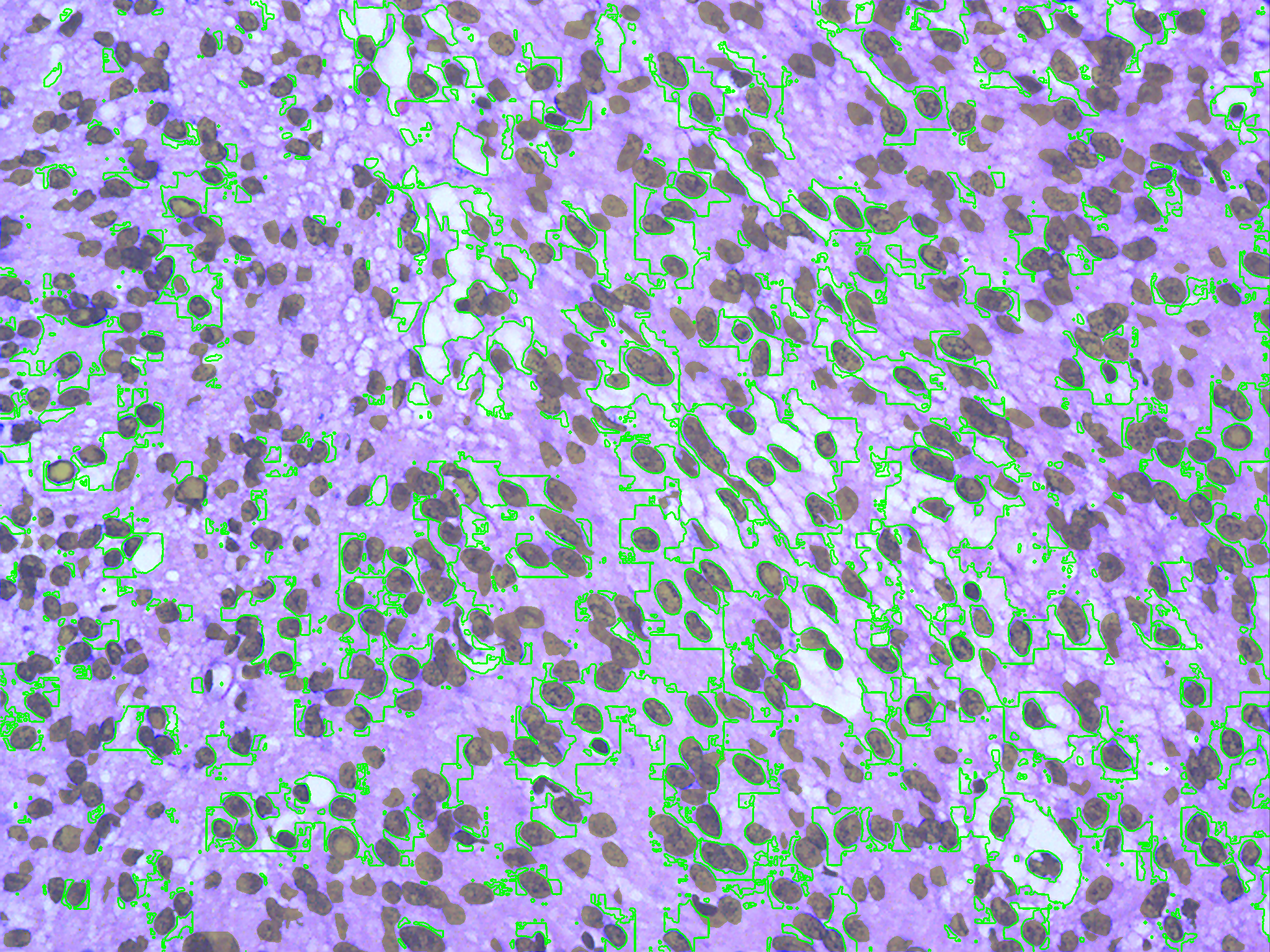

Supplement: S6 Fig — (ZIP) [file pone.0263006.s006.zip › Original U-net 2.jpg]

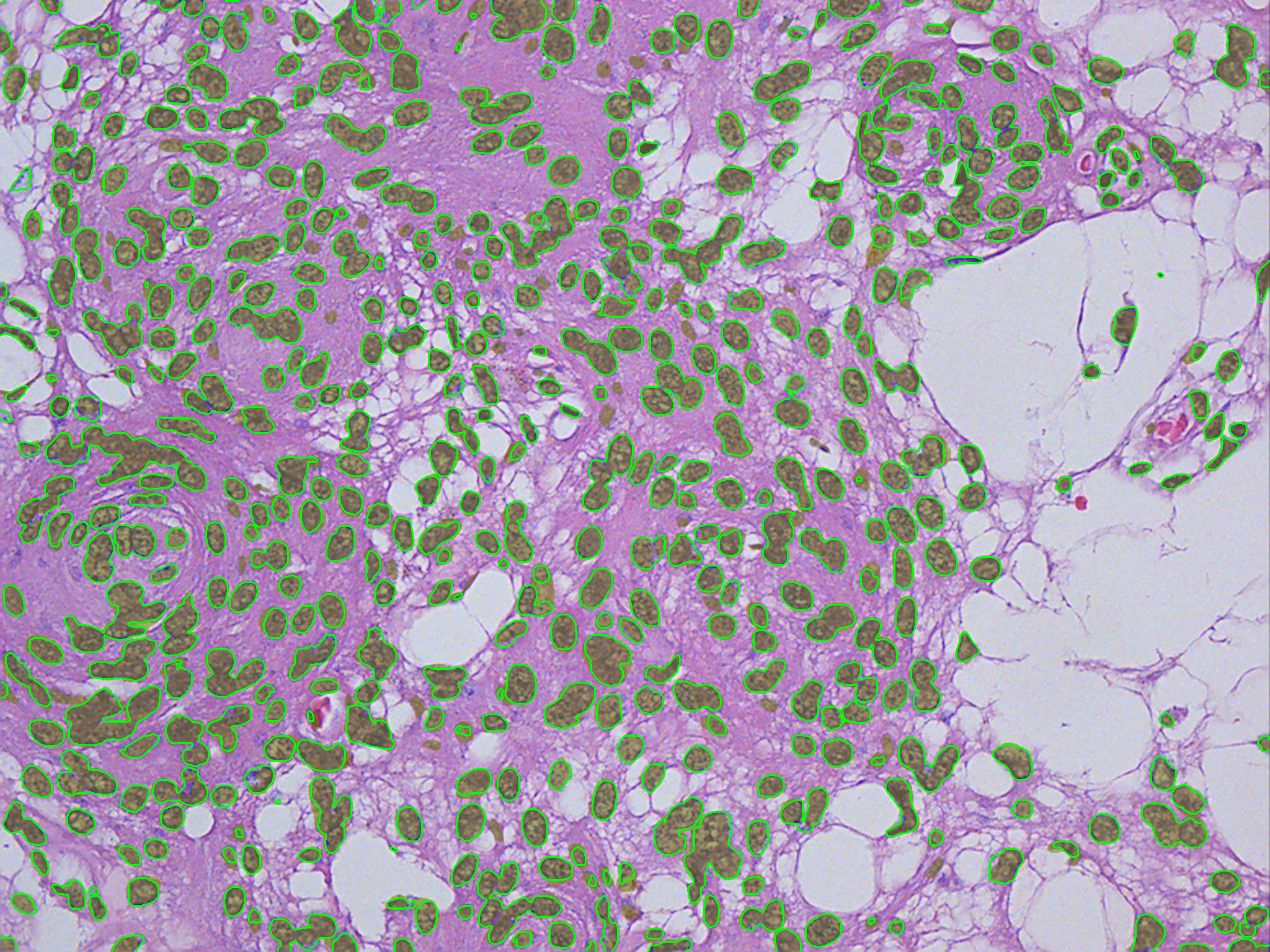

Supplement: S6 Fig — (ZIP) [file pone.0263006.s006.zip › Original U-net 3.jpg]
