# Supplementary material for: Segmentation of HE-stained meningioma pathological images based on pseudo-labels
Source: PLoS One. 2022 Feb 4;17(2):e0263006. doi: 10.1371/journal.pone.0263006 (PMC8815980; doi:10.1371/journal.pone.0263006)
Supplement: S1 File — (DOCX) [file pone.0263006.s007.docx]

File Fig 2:

Complete Image 1, Complete Image 2, Complete Image 3, and Complete Image 4 are the complete images corresponding to HE Image 1, HE Image 2, HE Image 3, and HE Image 4 of Figure 2 in the paper, respectively.

Original HE Image 1, Original HE Image 2, Original HE Image 3 and Original HE Image 4 are the original images corresponding to Complete Image 1, Complete Image 2, Complete Image 3 and Complete Image 4 of Fig 2 in the paper, respectively.

File Fig 3:

The Original HE Image is the original image corresponding to the HE Image in Fig 3 of the paper.

File Fig 4:

Original Image 1 and Original Image 2 are the original images corresponding to HE Image 1 and HE Image 2 of Fig 4 in the paper, respectively.

The Original Mixup Image is the complete image corresponding to the Mixed Image in Fig 4 of the paper.

Original Label 1, Original Label 2, and Original Mixup Label are the complete images corresponding to Label 1, Label 2, and Mixed Label of Fig 4 in the paper, respectively.

File Fig 5:

Original Low-grade Segmentation Result 1, Original Low-grade Segmentation Result 2, Original High-grade Segmentation Result 3 and Original High-grade Segmentation Result 4 are the complete images corresponding to 1、2、3、4 of Fig 5 in the paper, respectively.

Original Low-grade Image 1, Original Low-grade Image 2, Original High-grade Image 3, and Original High-grade Image 4 are the original images corresponding to HE Image 1, HE Image 2, HE Image 3, and HE Image 4 of Fig 5 in the paper, respectively.

File Fig 6:

Original HE Image 1, Original HE Image 2, and Original HE Image 3 are the original images corresponding to HE Image 1, HE Image 2, and HE Image 3 of Fig 6 in the paper, respectively.

Original K-means 1, Original K-means 2, and Original K-means 3 are the complete images corresponding to K-means 1, K-means 2, and K-means 3 of Fig 6 in the paper, respectively.

Original SVM 1, Original SVM 2, and Original SVM 3 are the complete images corresponding to svm 1, svm 2, and svm 3 of Fig 6 in the paper, respectively.

Original Ours 1, Original Ours 2, and Original Ours 3 are the complete images corresponding to ours 1, ours 2, and ours 3 of Fig 6 in the paper, respectively.

File Fig 7:

Original HE Image 1, Original HE Image 2, and Original HE Image 3 are the original images corresponding to HE Image 1, HE Image 2, and HE Image 3 of Fig 7 in the paper, respectively.

Original U-net 1, Original U-net 2, and Original U-net 3 are the original images corresponding to U-net 1, U-net 2, and U-net 3 of Fig 7 in the paper, respectively.

Original AttU-net 1, Original AttU-net 2, and Original AttU-net 3 are the complete images corresponding to AttU-net 1, AttU-net 2, and AttU-net 3 of Fig 7 in the paper, respectively.

Original R2U-net 1, Original R2U-net 2, and Original R2U-net 3 are the complete images corresponding to R2U-net 1, R2U-net 2, and R2U-net 3 of Fig 7 in the paper, respectively.

Original Ours 1, Original Ours 2, and Original Ours 3 are the complete images corresponding to Ours 1, Ours 2, and Ours 3 of Fig 7 in the paper, respectively.

File Fig 8:

Original HE Image 1, Original HE Image 2, and Original HE Image 3 are the original images corresponding to Complete segmention 1, Complete segmention 2 and Complete segmention 3 of Fig 8 in the paper, respectively.
