# Supplementary material for: Segmentation of HE-stained meningioma pathological images based on pseudo-labels
Source: PLoS One. 2022 Feb 4;17(2):e0263006. doi: 10.1371/journal.pone.0263006 (PMC8815980; doi:10.1371/journal.pone.0263006)
Supplement: Attachment — Submitted filename: Response to Reviewers.docx [file pone.0263006.s008.docx]

1. Please ensure that your manuscript meets PLOS ONE's style requirements, including those for file naming. The PLOS ONE style templates can be found at <https://journals.plos.org/plosone/s/file?id=wjVg/PLOSOne_formatting_sample_main_body.pdf> and <https://journals.plos.org/plosone/s/file?id=ba62/PLOSOne_formatting_sample_title_authors_affiliations.pdf>

Answer: The manuscript has been proofread and revised one by one according to the templates in the two links above.

2. Please provide additional details regarding participant consent. In the ethics statement in the Methods and online submission information, please ensure that you have specified (1) whether consent was informed and (2) what type you obtained (for instance, written or verbal, and if verbal, how it was documented and witnessed). If your study included minors, state whether you obtained consent from parents or guardians. If the need for consent was waived by the ethics committee, please include this information.

Answer: As claimed in Section II, the local ethics committee granted ethical approval for the study (Certificate No. 2019KJTYL024), and informed consent was obtained. The certificate is a formal document in Chinese, which was provided by Ethics Committee of Fujian Medical University. There was no minor included in this study, and all personal information had been hidden before the pathological images were used in this study.

3. We note that the grant information you provided in the ‘Funding Information’ and ‘Financial Disclosure’ sections do not match. When you resubmit, please ensure that you provide the correct grant numbers for the awards you received for your study in the ‘Funding Information’ section.

Answer: The grant numbers are double checked before filled into the ‘Funding Information’ section.

4. Thank you for stating the following in the Acknowledgments Section of your manuscript: This work was supported by the Fujian Science and Technology Innovation Joint Fund 466 (2018Y9112) and the Fujian Health Science Research Talent Training Project 467 (2019-ZQN-17). We note that you have provided additional information within the Acknowledgements Section that is not currently declared in your Funding Statement. Please note that funding information should not appear in the Acknowledgments section or other areas of your manuscript. We will only publish funding information present in the Funding Statement section of the online submission form. Please remove any funding-related text from the manuscript and let us know how you would like to update your Funding Statement. Currently, your Funding Statement reads as follows:

The funders had no role in study design, data collection and analysis, decision to publish, or preparation of the manuscript.

Please include your amended statements within your cover letter; we will change the online submission form on your behalf.

Answer: Thanks for the reminder. We have removed the specific funding information from Acknowledgements section. We also included the above Funding Statement into the revised cover letter following your instructions.

5. In your Data Availability statement, you have not specified where the minimal data set underlying the results described in your manuscript can be found. PLOS defines a study's minimal data set as the underlying data used to reach the conclusions drawn in the manuscript and any additional data required to replicate the reported study findings in their entirety. All PLOS journals require that the minimal data set be made fully available. For more information about our data policy, please see <http://journals.plos.org/plosone/s/data-availability.>

Upon re-submitting your revised manuscript, please upload your study’s minimal underlying data set as either Supporting Information files or to a stable, public repository and include the relevant URLs, DOIs, or accession numbers within your revised cover letter. For a list of acceptable repositories, please see <http://journals.plos.org/plosone/s/data-availability#loc-recommended-repositories.> Any potentially identifying patient information must be fully anonymized.

Important: If there are ethical or legal restrictions to sharing your data publicly, please explain these restrictions in detail. Please see our guidelines for more information on what we consider unacceptable restrictions to publicly sharing data: <http://journals.plos.org/plosone/s/data-availability#loc-unacceptable-data-access-restrictions.> Note that it is not acceptable for the authors to be the sole named individuals responsible for ensuring data access. We will update your Data Availability statement to reflect the information you provide in your cover letter.

Answer: We have uploaded the minimal underlying data set and the related specification as Supporting Information files, including all original images which were processed and shown in the figures of the manuscript. Our research group is also building a full test dataset used in the experiments of the manuscript, which is under reviewing by the local ethics committee. The dataset will be uploaded online to public once the application is approved. Before putting the whole dataset online, all readers can ask for original images directly by contacting the corresponding author.

6. PLOS requires an ORCID iD for the corresponding author in Editorial Manager on papers submitted after December 6th, 2016. Please ensure that you have an ORCID iD and that it is validated in Editorial Manager. To do this, go to ‘Update my Information’ (in the upper left-hand corner of the main menu), and click on the Fetch/Validate link next to the ORCID field. This will take you to the ORCID site and allow you to create a new iD or authenticate a pre-existing iD in Editorial Manager. Please see the following video for instructions on linking an ORCID iD to your Editorial Manager account: <https://www.youtube.com/watch?v=_xcclfuvtxQ>

Answer: Thanks for your kind reminder. The ORCID of the corresponding author, Dr. Peng Shi, is 0000-0001-7124-0210, which has been included in the revised submission.

7. Please review your reference list to ensure that it is complete and correct. If you have cited papers that have been retracted, please include the rationale for doing so in the manuscript text, or remove these references and replace them with relevant current references. Any changes to the reference list should be mentioned in the rebuttal letter that accompanies your revised manuscript. If you need to cite a retracted article, indicate the article’s retracted status in the References list and also include a citation and full reference for the retraction notice.

Answer: The format of references in the manuscript has been corrected in accordance with the format in the template. Due to the need for manuscript revisions, references 28 to 31 have been added.

**Review Comments to the Author**

Reviewer #1: The proposed method and completed experiments in this paper are in line with my expectations for a manuscript on medical image processing. It would be better if there were an accepted dataset to support the experiment, can the authors provide such a supplement for this paper? In addition, some grammatical spelling may need to be a little more careful.

Answer: Thank you for your helpful comments on the manuscript. In response to this problem, we tested the performance of the proposed method on the MoNuSeg dataset, The MoNuSeg dataset comes from the Medical Image Computing and Computer Assisted Intervention (MICCAI) 2018 Multi-Organ Pathology Image Nucleus Segmentation Challenge, which includes 30 training sets and 14 test sets. The detailed results are shown in Section Ⅲ.

Reviewer #2: 1. No statistical description of the validity of the method was seen, suggesting significance.2. Comparison of images can be compared with other methods to prove the advantages of this method. The image is very good.

Answer: Thank you for your suggestions, they are very helpful. For this, we selected 150 high-grade and low-grade meningiomas with HE staining pathology images, a total of 300 images, and performed cell nucleus segmentation according to the proposed method and counted relevant pathological features. Then, the two groups of features of pathological images of high- and low-grade meningiomas were tested for differences to verify the auxiliary effect of pathological features based on the segmentation results on meningioma grading. Details can be found in Section Ⅲ.

Since the meningioma dataset used only has nucleus foreground annotations and no internucleus segmentation annotations, the comparison with other methods can only give a comparison chart of the nucleus foreground segmentation results, as shown in section Ⅲ.
